# Trans-omics approaches used to characterise fish nutritional biorhythms in leopard coral grouper (*Plectropomus leopardus*)

**DOI:** 10.1038/s41598-017-09531-4

**Published:** 2017-08-24

**Authors:** Miyuki Mekuchi, Kenji Sakata, Tomofumi Yamaguchi, Masahiko Koiso, Jun Kikuchi

**Affiliations:** 1 RIKEN Center for Sustainable Resource Science, 1-7-22 Suehiro-cho, Tsurumi-ku, Yokohama, Kanagawa 230-0045 Japan; 2 National Fishery Research Institute of Fishery Sciences, Fishery Research and Education Organization, 2-12-4, Fukuura, Kanazawa-ku, Yokohama, 230-0045 Japan; 3 Research Center for Subtropical Fisheries, 148 Fukaiota, Ishikagi, 907-0451 Japan; 4 Graduate School of Medical Life Science, Yokohama City University, 1-7-29 Suehirocho, Tsurumi-ku, Yokohama, Kanagawa 230-0045 Japan; 5 Graduate School of Bioagricultural Sciences, Nagoya University, 1 Furo-cho, Chikusa-ku, Nagoya, Aichi 464-0810 Japan

## Abstract

Aquaculture is now a major supplier of fish, and has the potential to be a major source of protein in the future. Leopard coral groupers are traded in Asian markets as superior fish, and production via aquaculture has commenced. As feeding efficiency is of great concern in aquaculture, we sought to examine the metabolism of leopard coral groupers using trans-omics approaches. Metabolic mechanisms were comprehensively analysed using transcriptomic and metabolomic techniques. This study focused on the dynamics of muscular metabolites and gene expression. The omics data were discussed in light of circadian rhythms and fasting/feeding. The obtained data suggest that branched-chain amino acids played a role in energy generation in the fish muscle tissues during fasting. Moreover, glycolysis, TCA cycles, and purine metabolic substances exhibited circadian patterns, and gene expression also varied. This study is the first step to understanding the metabolic mechanisms of the leopard coral grouper.

## Introduction

Fish and fishery products are valuable sources of proteins and essential nutrients, and they provide human health benefits and facilitate environmental sustainability^1–3^. Global fish production from aquaculture has increased over the past few decades, while that from capture fisheries has decreased slightly^4–6^. Aquaculture has the potential to produce substantial amounts of fish in the future to support increases in human population size. In addition, aquaculture also allows suitable responses to customer demands, such as improved safety and quality. According to an FAO report, more than 50% of food fish consumed globally will eventually be produced by aquaculture^7^, and aquaculture is now the main source of increased fish supplies. Feed is one of the largest production costs associated with aquaculture, and the price of fishmeal is predicted to increase as aquaculture expands worldwide. Therefore, feed efficiency improvement is required, and feeding concerns will be major factors limiting aquaculture development.

**Figure 1:**
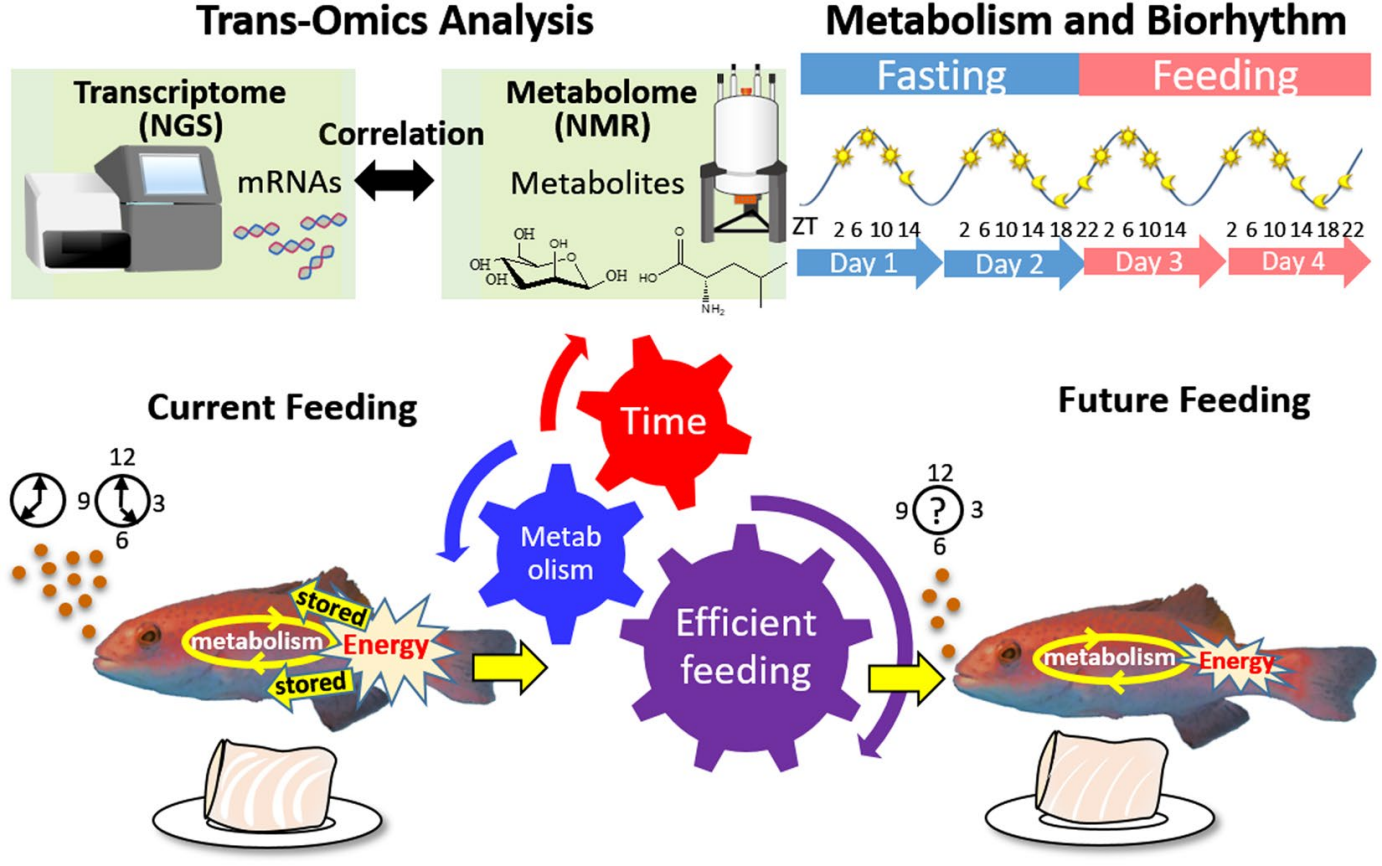
Schematic diagram of this study. Transcriptome analysis by next-generation sequencing and metabolome analysis by NMR were conducted. Leopard coral grouper were reared in fasting for 2 days and sequentially in feeding for 2 days. Samples were collected periodically. Time is showed in Zeitgeber time (ZT). Correlation based integrated analysis revealed that genes and metabolites contributed to the circadian rhythm and the energy-yielding nutrients condition. Currently, excess amount of energy derived from excess amount of feeding is stored in the muscle and vesicle fat. Our metabolic profile data in the consideration of circadian rhythm would help for improvement of feeding techniques by finding out the efficient time and the amount of feeding. Fishes were drawn using the free software GIMP (https://www.gimp.org) based on the photograph taken by Miyuki Mekuchi. NGS and NMR were drawn by Kenji Sakata.

Feed efficiency is inextricably linked to the metabolic and biorhythmic characteristics of the cultured fish; thus, to better understand the fundamental mechanisms of metabolic and biorhythmic processes in aquaculture, we focused on global analyses based on gene expression and metabolic and biorhythmic dynamics (Fig. 1). Trans-omics approaches^8^, including the integration of transcriptome and metabolome analyses, inform the comprehensive data network of genes and metabolites^9–16^. The combination of these high-throughput technologies revealed holistic and multidimensional information associated with various functions and pathways.

The leopard coral grouper, *Plectropomus leopardus*, has recently become an important commercial species worldwide and is currently trading at a high price^17^. As increasing demands, supply of farmed fish has been required. Aquaculture technology associated with leopard coral groupers has been developed; however, there are still differences between wild and artificial fish (Fig. S1). Aquaculture technology needs to be improved in the areas of feed efficiency and metabolic control. Digestion and absorption have been studied in select species over the past two to three decades. However, metabolic mechanisms differ among species, and to our knowledge, there are no current reports about this trait in grouper species. Recently developed analysis-based techniques allow the execution of comprehensive studies of metabolic mechanisms. Our purpose of this study is to understanding the metabolic mechanisms of the leopard coral grouper muscles, which are known to be sites of gluconeogenesis^18^. This study is a data-driven approach and the scheme is shown in Figure S2. Our metabolic profile data in the consideration of circadian rhythm would help developing feeding technology.

## Results

### *De novo* transcriptome assembly of leopard coral grouper muscle tissues

The results of the transcriptomic analysis were used to describe responses to feed intake and associated circadian rhythm characteristics. The constructed cDNA libraries from leopard coral grouper muscle samples were sequenced using an Illumina NextSeq 500. An average of approximately 7.9 million sequence reads was obtained from each individual sample. For de novo assembling, 331 million reads were initially subjected to trimming for the removal of low quality and ambiguous nucleotides. Clean reads (328.6 million reads) were left and used for *de novo* assembling by CLC Genomics Workbench 8.1. (https://www.qiagenbioinformatics.com/). The *de novo* assembly contained 86,790 contigs that were greater than 200 bp. To identify putative functions, the 86,790 contigs were aligned to the Ensembl zebrafish protein database using BLASTX. The BLASTX results were sorted based on the zebrafish protein ID, and several contigs shared the same ID. Therefore, the contig sequences with the highest e-values were selected as representatives. A total of 19,737 selected contigs were selected as reference sequences to calculate the reads per kilobase of exon model per million mapped reads (RPKM) expression values. RPKM values were used to establish the total number of genes expressed in the muscle transcriptome, and 80% of the reads were mapped to the reference contigs. For further differential expression analyses, only transcripts with a normalised RPKM > 5 for each sample were considered. Selected genes from the Figure S5 were analysed by quantitative PCR to validate RNA-seq (Fig. S6). The mRNA expression pattern obtained using quantitative PCR showed the same tendency to RNA-seq data. Overall, the highest mRNA expression levels in the leopard coral grouper muscle were associated with myosin, followed by parvalbumin, actin, tropomyosin and creatine kinase, muscle type (Table S1).

### Gene ontology and hierarchical clustering analysis

Gene ontology (GO) terms were assigned, and 13,960 contigs were categorised based on associated biological processes, molecular functions, and cellular components. Molecular function terms represent the biochemical activities. The major categories in molecular function were catalytic activity (36.1%) and binding (35.4%) (Fig. S3A), Biological process terms is defined as the biological goals that the gene or gene product contributes. In this category, genes contributed to cellular process were 41.7% and metabolic process were 34.6% (Fig. S3B). Cellular component refers to locations that a gene product is active. 38.4% of genes were localized in cell part, 22.3% were in organelle, 17.2% were in membrane (Fig. S3C).

**Figure 2:**
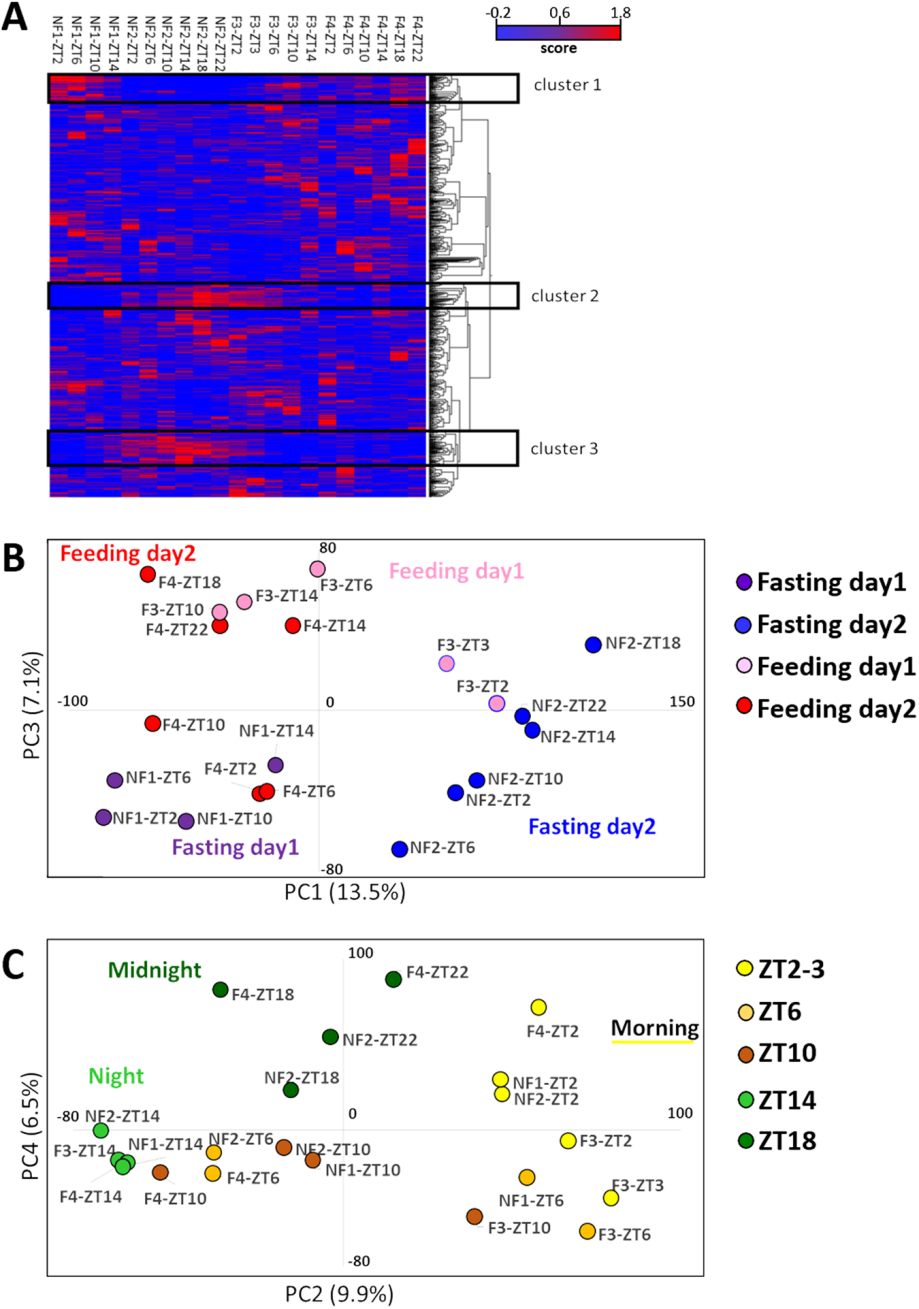
Transcriptome analysis in fish muscle. (**A**) Heat map with hierarchical clustering analysis. First day of experiment without feeding (NF1), second day without feeding (NF2), third day with feeding (F3), fourth day with feeding (F4). ZT stands for Zeitgeber time. Red column showed the levels of expression were high, on the other hand, blue showed the levels were low. The result of clustering analysis was illustrated by tree-like categorizations, which exhibited the organizing information regarding the similarity. Three distinct clusters were identified; the levels of expression were low in fasting (cluster 1), high in late fasting (cluster 2), high in early fasting (cluster 3). (**B**) The clusterisation of feeding and fasting by PCA analysis. PCA analysis displayed the difference between fasting and feeding. The graph was constructed using PC1 and PC3. The highest levels of nutrient depletion (NF2 and F3 ZT2-3) were located on the positive side of PC1. (**C**) The result of PCA analysis using PC2 and PC4. Morning cluster was located on PC2 positive side; on the other hand, night clusters were located on the negative side of PC2.

A cluster heat map was constructed to characterise the data and to provide useful insight into the mRNA expression dynamics. For hierarchical clustering analyses (HCA), the Euclidean distance was utilised to measure the distance, and clustering was conducted using the complete linkage analysis method. The results of the HCA confirmed several clusters, and distinct dynamic patterns that varied with short-term fasting were observed (Fig. 2A). Transcripts belonging to these clusters were selected, and gene ontology analyses were subsequently performed (Table S2). The three selected clusters in the heat map corresponded to the following: (Cluster 1) low expression levels during the fasting stage; (Cluster 2) upregulated expression levels during the early fasting stage; and (Cluster 3) upregulated expression levels during the late fasting condition. Regarding metabolism, the proportion of carbohydrate and cellular amino acid metabolism characteristics differed among clusters (Fig. S4A–C). The results of Clusters 2 and 3 were similar to those of Cluster 1, and the tricarboxylic amino acid cycle category was only detected in Cluster 1.

### Sample profiling using a principal components analysis

A principal components analysis (PCA) was performed in order to compare the profiles of the sequenced transcripts, and the data were projected to PC1 and PC3 (Fig. 2B). We identified the following four subpopulations based on differences in time-course dependence: fasting day 1 (negative side of PC1); fasting day 2 (positive side of PC1); feeding day 1 (positive side of PC3); and feeding day 2 (negative side of PC1). The fasting stage represented the stage with the highest levels of nutrient depletion (NF2 and F3 ZT2-3), and it was clearly distinguished based on its location on the positive side of PC1. Regarding PC2 and PC4, we identified clusters that were dependent on the time of the day (Fig. 2C). For instance, the morning cluster (ZT2) was located on the positive side of PC2, but the night cluster (ZT14) was located on the opposite side of the morning cluster. Moreover, the midnight cluster was located on the positive side of PC4, and the daytime cluster was located on the opposite side of the midnight cluster. PC1 accounted for 13.5% of the variation, and PC2, PC3, and PC4 accounted for 9.9%, 7.1%, and 6.5% of the variation, respectively.

### Gene expression comparisons

#### Circadian rhythm

Sequential expression pattern were analysed. Initially, the dynamics of the molecular core clock were examined^19^. In leopard coral grouper muscle, four period circadian clock (*per*) mRNAs were found to be expressed. The gene expression patterns of *per1b* and *per3* exhibited circadian rhythms (Fig. S5); however, *per1a* and *per2* did not show a rhythmic pattern and their levels of gene expression were considerably lower than those of *per1b* and *per3*. Cryptochrome circadian clock (*cry*) also contained four genes; *cry1aa*, *cry1ab*, *cry1ba*, and *cry2*. All four genes exhibited circadian rhythms. Clock circadian regulator (*clock*) had two kinds of genes. *Clocka* showed a circadian pattern, while the expression level of *clockb* was considerably lower. CLOCK-interacting pacemaker (*cipc*) was also found to exhibit 24-h rhythms. Since circadian rhythm is known to involve growth hormone expression^20^, we examined the expression pattern of growth hormone-related genes. The growth hormone receptor (*ghr*) a and b showed circadian patterns. *Per1b*, *per3*, *cry1ab*, and *cry2* were found to have high expression in the morning (ZT2). The expression of *cry1aa* was high during the day (ZT6). *Cry1ba* and *clocka* expression was high in the afternoon (ZT10). The expression of *cipc* was high at night (ZT14-22). We further analysed the gene expression pattern of all transcripts displaying the same patterns as the circadian-patterned genes. The results are shown in Figure S5. Several muscle protein-related genes were found to have identical expression patterns to those of the circadian core genes. Moreover, myogenic differentiation 1 (*myoD*) and myogenin (*myog*) were found to have a correlation with clock genes (Fig. S5).

#### Feeding and Fasting

A large number of mRNA transcripts significantly differed between the fasting and feeding stages. The expression data of fasting day 2 and feeding day 2 were compared (Table S3A–F; S3A for ZT2, S3B for ZT6, S3C for ZT10, S3D for ZT14, S3E for ZT18, and S3F for ZT22). For instance, 2382 mRNA transcripts that satisfied the criteria of *p-*value < 0.05 and fold-change ≥ 2.0 were utilised for further analysis. During the feeding stage, 984 and 1398 genes were upregulated and downregulated, respectively, as compared to the fasting stage. The largest differences between the expression levels during the fasting and feeding stages were calcium-binding and coiled-coil domain-containing proteins, which were 131.64 times higher during the fasting stage (Table S3B). Uncharacterised proteins were 58.64 times higher during the fasting stage (Table S3B), followed by N-lysine methyltransferase SMYD2-A-like, which was 20.36 times higher (Table S3E). GO analyses were also performed, focusing on metabolism (Fig. S7). For instance, glycolysis-related genes were upregulated from ZT10 to ZT18, and glyconeogenesis-related genes were found only in downregulated groups (Table S3A-E). The number of protein metabolic process and nucleobase-containing compound metabolic process genes differed significantly throughout the day. The gene expression levels of fatty accumulation and hormones related to juvenile growth were examined. Adiponectin receptor2 (*adipoR2*), growth hormone receptor b (*ghrb*), thyroid hormone receptor alpha (trα), and thyroid hormone receptor interactor 11(*trip11*) were found to changed during the feeding and fasting (Fig. S5).

### Metabolomic analysis of leopard coral grouper muscle via NMR

Metabolic profiles were evaluated using an NMR-based metabolomic analysis that examined water-soluble extracts of the leopard coral grouper muscle samples. The annotated metabolites from the samples were characterised using HSQC and HSQC-TOCSY (Fig. S8A and B, Table S5), and a two-dimensional (2-D)^1^H J-resolved spectroscopy spectrum was used to quantify the metabolites (Fig. S9). This method identified the chemical shifts and J-couplings of the metabolite and facilitated metabolite identification and quantification. The 2-D spectrum was simplified and used calculated 1-D projections followed by multivariate analysis.

**Figure 3:**
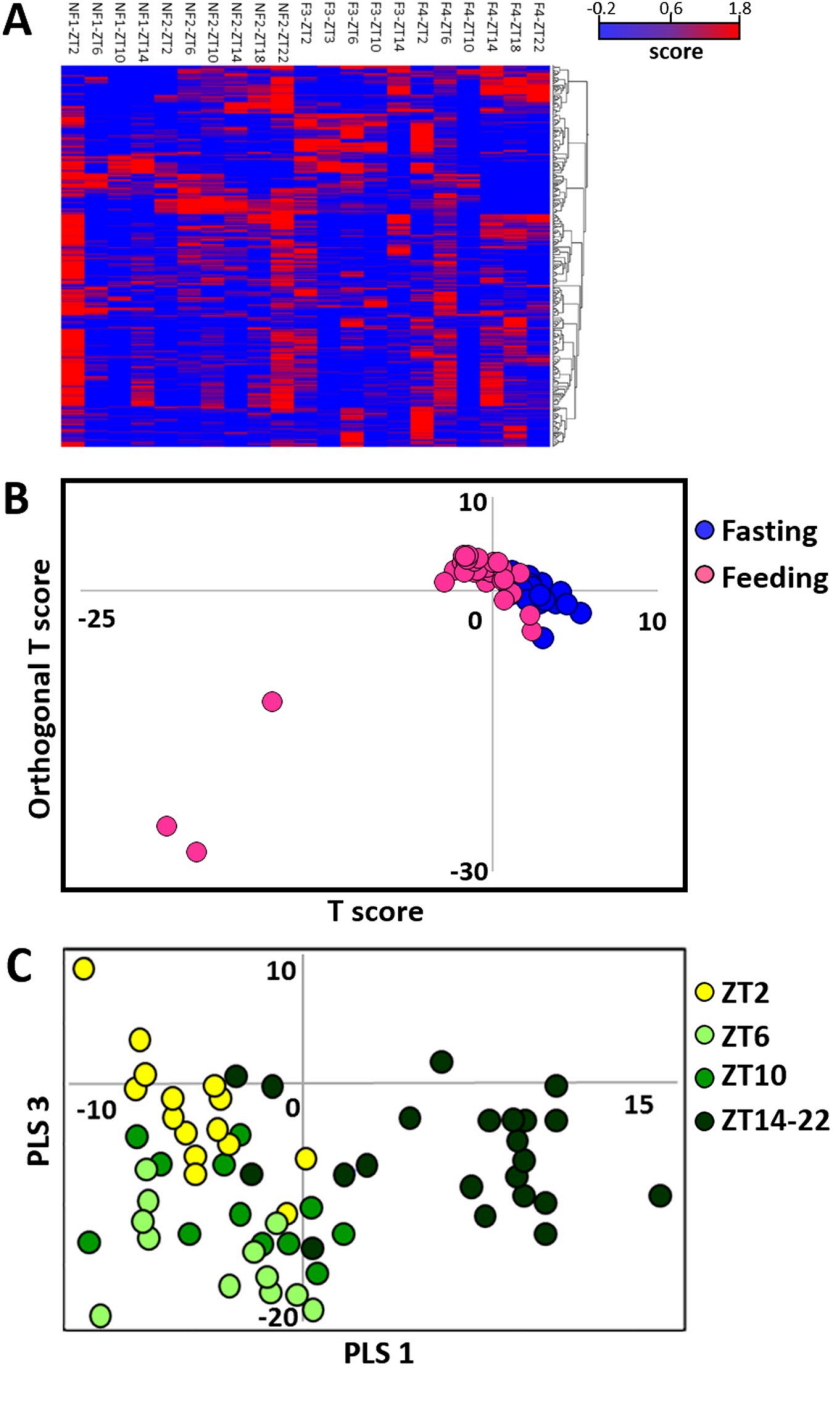
Metabolome analysis by NMR in fish muscle. (**A**) Heat map with hierarchical clustering analysis. Red showed the amount of metabolites was high, on the contrary, blue showed the low levels. (**B**) The result of a discrimination analysis on a nutritional condition by OPLS-DA. Feeding cluster is located on the top left direction. Fasting cluster is located on the bottom right area. (**C**) The discrimination analysis by PLS-DA. Clusters were formed by time dependent manner. Yellow circles show ZT2 cluster, pale green show ZT6 cluster, green show ZT10 cluster, and dark green show night cluster, ZT14-AT22.

A total of 407 peak signals were quantified based on their spectral intensities. In particular, the following six distinct high-intensity peaks were observed: lactate (1.34 ppm), creatine (3.02 ppm, 3.92 ppm), trimethylamine oxide (TMAO; 3.25 ppm), taurine (3.41 ppm), and glycine (3.53 ppm). Multivariate analyses were performed to characterise the obtained data, and the dynamics of the metabolite changes were observed using the^1^H J-resolved spectroscopy spectrum. A cluster heat map was constructed to explore complex metabolomic data (Fig. 3A), and the metabolomic heat map aided in the identification of clusters from the sample sets and the analysis of the metabolite features. The OPLS-DA score plot demonstrated that the metabolic profiles were also likely to cluster based on differences between the feeding and fasting stages (Fig. 3B). For instance, the feed group is located on the negative side of T score. Moreover, PLS-DA indicated the variation between day and night samples (Fig. 3C), and the night group is clustered on the positive side of PLS1. The loading plots of OPLS-DA and PLS-DA revealed that metabolites such as inosine, leucine, isoleucine, and inositol monophosphate (IMP) contributed to the discrimination profiles (Fig. S10A,B).

### Integration analysis of transcriptome and metabolome data

**Figure 4:**
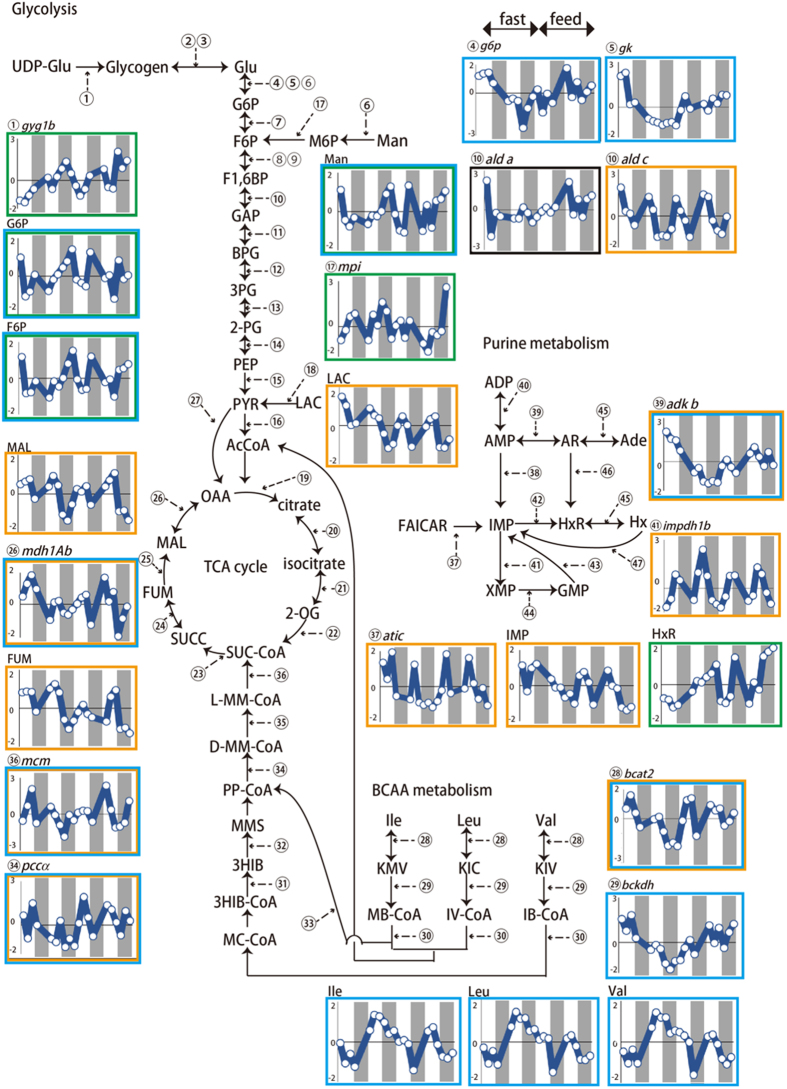
A schematic chart of glycolysis and tricarboxylic acid cycles. The metabolites and gene expression exhibited the characteristic dynamics is showed in the diagram. Yellow colored gene and metabolites exhibiting circadian fashion and expressed high in the day and morning. Green showed nocturnal fashion. Blue and pink showed the upregulation by fasting and feeding, respectively. Dark colored areas represent night. Numbers within a circle indicated the genes. The detailed gene expression profiles were showed in Figures S9–S11. Glycogenin synthase 1b (*gyg1b*), glucose-6-phosphate dehydrogenase (*g6p*), glucokinase (*gk*), aldolase (*ald*), phosphate isomerase (*mpi*), malate dehydrogenase (*mdh*), branched chain aminotransferase2 (*bcat2*), branched chain keto acid dehydrogenase (*bckdh*), propionyl-CoA carboxylase (*pcc*), methylmalonyl-CoA mutase (*mcm*), IMP cyclohydrase (*atic*), adenosine kinase b (*adk b*), IMP dehydrogenase 1b (*impdh 1b*) glucose (Glu), glucose-6-phosphate (G6P), fructose 6-phosphate (F6P), mannose-6-phosphate (M6P), mannose (Man), fructose-1,6-biphosphate (F1,6BP), glyceraldehyde-3-phosphate(GAP), 1,3-bisphosphoglycerate (BPG), 3-phosphoglyceric acid (3PG), 2-phosphoglycerate (2PG), phosphoenolpyruvic acid (PEP), pyruvic Acid (PYR), acetyl-CoA (AcCOA), oxaloacetic acid (OAA), 2-oxoglutarate (2-OG), succinyl-CoA (SUC-CoA), succinic acid (SUCC), fumarate (FUM), malate (MAL), L-methylmalonyl CoA (L-MMCoA), R-methylmalonyl CoA (R-MMCoA), propionyl-CoA (PP-CoA), methylmalonate-semialdehyde (MMS), 3-hydroxy-isobutyrate (3HIB), 3-hydroxy-isobutyrate CoA (3HIB CoA), methacrylyl-CoA (MC-CoA), a-ketoisocaproate (KIC), a-ketoisovalerate (KIV), a-keto-s-methylvalerate (KMV), isovaleryl-CoA (IV-CoA), isobutyryl-CoA (IB-CoA), a-methylbutyryl-CoA (MB-CoA), valine (Val), leucine (Leu), isoleucine (Ile), adenosine monophosphate (AMP), adenosine diphosphate (ADP), succinyl AMP (SucAMP), adenosine (AR), adenine (Ade), inosine monophosphate (IMP), Inosine (HxR), hypoxanthine (Hx), xanthosine monophosphate (XMP), guanosine monophosphate (GMP), and 5-form-aminoimidazole-4-carboxamide ribonucleotide (FAICAR).

To understand the dynamics of metabolites and mRNA transcripts that varied in a circadian fashion and in response to food intake, a sequential pattern search based on correlation analyses with a teacher dataset was performed using transcriptome and metabolome data (Fig. 4). The scheme of study was shown in Figure S2. These results corroborated the results of the PCA and the discrimination analysis. For example, lactate, malate, fumarate, and IMP exhibited 24-h rhythmicity, and the amounts were highest in the daytime. The symmetric pattern of those metabolites was fructose 6-phosphate (F6P), glucose 6-phosphate (G6P), mannose, and inosine (HxR). The amount of isoleucine, leucine, and valine was high in the fasting, on the other hand, F6P, G6Pand mannose was low in the fasting. With regard to metabolism, genes related to purine metabolism, glutamine metabolism, and carbohydrate metabolism were found (Fig. S11–S13).

## Discussion

Transcriptome analyses revealed the protein constitution of coral leopard grouper muscle tissues. Most of the transcripts consisted of myosin, parvalbumin, and actin genes (Table 1). These three genes accounted for approximately 80% of all transcripts. Myosin is the most essential component of muscle tissue, and the heavy chain subunit of myosin plays an important role in the contraction of muscles using ATP^21^. Parvalbumins are the abundant calcium-binding proteins in fast-contracting muscles^22^, and actin is a major cytoskeletal protein that is involved in cell motility, structure, and integrity. Moreover, the interaction of actin with myosin results in muscle contractions^23^. There have been several studies on transcriptome analysis of teleostean muscle^24–29^. Our GO analysis results exhibited similar trends to those observed in previous studies^24^. For biological processes, the major GO terms were cellular process and metabolic process, although the accurate proportion was not consistent.

With regard to the gene expression of circadian rhythm, PCA can be used to identify fundamental temporal patterns. Regarding the projection of PC2 and PC4, the data were divided into four main categories based on association with day and night (Fig. 2C). The expression patterns of circadian core genes were initially examined to identify genes whose expression patterns showed 24-h rhythms^19, 30^. *Per1a*, *per3*, *cry1aa*, *cry1ab*, *cry1ba*, *cry2*, *clocka*, and *cipc* genes showed circadian rhythm (Fig. S5). Compared to previous studies on zebrafish and Chinese perch skeletal muscle, *per1b* and *per3* in leopard coral grouper showed the same expression patterns as those in zebrafish; however, the expression levels in perch *per* were higher, at around ZT21. *Clock* expression showed similar, but not identical patterns^31^. The expression patterns of *cry* in the present study differed from those of zebrafish^32^. These core genes are known to be involved in the perception of time in the pineal gland and central nervous system; however, the present study revealed that these genes are also expressed in skeletal muscle and show circadian patterns. This result indicates that muscle has its own system of recognising 24-h rhythms and that mRNA expression patterns differ among species. Among the core genes, there were no genes whose expression pattern varied according to nutritional condition. Among myosin-related genes, *myoD* showed a 24-h rhythm (Fig. S5). The highest expression levels of myogenin and *myoD* occurred at ZT6 and ZT2, respectively. The expression pattern of myogenin was identical to that of zebrafish in a previous study; however, *myoD* expression patterns differed from those in previous studies^32^. MyoD and myogenine are known to the myogeninc regulatory factors (MRFs)^33^. Murine CLOCK and BMAL1 need to control *MyoD* and keep the phenotype and functions of the skeletal muscle^34^.

Correlation-based analyses were performed to find genes exhibiting the same expression patterns as the circadian core genes (Fig. S5). Growth hormone secretion has been known to follow circadian patterns^35^. Growth hormone genes did not follow a circadian pattern; however, growth hormone receptor exhibited a 24-h pattern. Correlation analysis was applied to identify genes displaying the same circadian pattern as growth hormone receptor. Among the growth hormone-related genes, growth hormone receptor was found to exhibit a circadian pattern. The gene expressions of thyroid hormone receptor a (*trα*) and thyroid hormone receptor interactor 11(*trip11*) were upregulated by fasting. Hormones on the hypothalamic–pituitary–thyroid axis (HPT axis) play important roles in growth and metabolism^36^. In skeletal muscle, the thyroid hormone receptor α gene has been reported to be more highly expressed than β^37^. It was predicted that *trip11* would interact with *trα*, as the dynamics patterns of those two genes are similar. Adiponectin receptor 2 (*adipoR2*) showed the same expression pattern, and its expression levels were high during the night (Fig. S5). Adiponectin and its receptors are important substances in controlling the amount of body fat^38^. In leopard coral grouper muscle, three kinds of adiponectin receptor were found. The expression levels of muscular adiponectin receptor were low, particularly for 1a and 1b. These results were different from those found in other species^39^.

The results of metabolome analysis via NMR were used in subsequent multivariate analysis. In circadian rhythm, PLS-DA was conducted to find the metabolites of characteristic contributors (Fig. 3C). Samples were divided into four groups (ZT2, ZT6, ZT10, and ZT14-22 [night]). The results of the loading plot analysis suggested that inosine (HxR) was a contributor to the positive side of Comp1 and characterised the night group (Fig. S10B). The biggest factor that separated the morning group was located on the negative side of PLS1, and the results of the loading plot analysis revealed that IMP, which was high in the morning, was a dominant contributor. A similar dynamic pattern was observed in the zebrafish brain^40^. IMP and inosine are ATP-related compounds that are important components of the energy production process, and IMP is generated by removal of the amine group from AMP^41^. Inosine is generated by the deamination of adenosine or via the reaction between 5′-nucleotidase and IMP. Nucleotidase is a hydrolytic enzyme that aids in the conversion of IMP to inosine. Although many kinds of nucleotidase genes were found, they exhibited different expression tendencies. On the negative side of PLS1, the morning (ZT2) and evening (ZT6-10) groups were discriminated by the degree of PLS3 (Fig. 3). The results of the loading plot analysis found that malate, fumarate, and lactate, which are associated with TCA cycle and glycolysis^42, 43^, were contributors. These three substances were high in the evening and low at night. The dynamic pattern analysis, based on correlation tests, revealed that lactate, malate, and fumarate were associated with diurnal 24-h rhythmicity pattern. Lactate in the muscle exhibited the same dynamics pattern as the zebrafish^40^. In medaka liver, the amount of malate and fumarate decreased in night time^44^. TCA cycle and glycolysis is a major pathway for the generation of ATP, which is thought to be consumed during the day than at night. On the other hand, inosine, mannose, G6P, and F6P displayed nocturnal 24-h rhythmicity, and mannose, G6P, and F6P are also intermediate products of ATP generation pathways. It has been reported that glucose-related compounds are affected by genes associated with circadian rhythms^45^. Regarding mRNA transcripts, glycogenin was found to have nocturnal 24-h rhythmicity. The level of ATP consumption is low at night, so excess amounts of sugar are thought to be converted to the glycogen storage form via the use of glycogenin. Moreover, mannose is utilised during glycolysis, and G6P and F6P are intermediate products of glycolysis^46^. The ATP consumption rate was slow during the night, so the intermediate products accumulated as the reaction speed of the TCA cycle decreased.

Regarding the gene expression in fasting and feeding, the projection of PC1 and PC3 of the PCA analysis indicated a temporal pattern associated with feed intake (Fig. 2B), and the nutrient depletion group was located on the positive side of PC1. Specific mRNA expression patterns were also detected from the heat map (Fig. 2A), and the expression pattern of Cluster 1 was low on Day 2 during nutrient depletion. On the other hand, Clusters 2 and 3 were high on Day 2 during nutrient depletion. Compared to Cluster 2, the time of increased expression was earlier in Cluster 3. Moreover, genes in the three clusters were subjected to gene ontology analysis (Fig. S3). Regarding the biological processes, the component (in the metabolic category) proportion varied among the three clusters. The number of monosaccharide-related genes increased in Cluster 1, but the number of polysaccharide-related genes decreased (data not shown). Polysaccharide is the sugar form that is present before digestion, and is used as source of energy. The number of polysaccharide-related genes was reduced in response to the fasting stage, which suggests that the polysaccharides derived from feed are already digested during the fasting period. Regarding the protein metabolic process, proteolysis increased during the fasting stage, and this suggests that amino acids were produced from protein during the fasting stage (data not shown). These amino acids are thought to be used for energy production. Furthermore, the mRNA expression levels from experiment Day 2 (the second fasting day) and Day 4 (the second feeding day) were compared for every sampling point, and GO analysis was performed focused on metabolic process (Fig. S7). Gluconeogenesis-related genes were only observed in the downregulated group (Feed <Fast), which implies that gluconeogenesis actively occurred during fasting in order to generate energy. It is well known that glucose is synthesised from non-carbohydrate substances to generate energy in teleostean skeletal muscle^47^. Furthermore, several glycolysis-related genes were upregulated, suggesting that energy was generated from glucose derived from feeding. Feeding and fasting studies in fish muscle have been reported^28, 29, 48, 49^. The levels of parvalbumin were higher for feeding than fasting. This result showed a similar trend to that observed for sea bream^48^. However, the expression levels of GAPDH showed no change during the experiment. The expression of slow myosin heavy chain 2 has been reported to be high in fasting^48^; however, our study did not show the same trend as a previous study. We focused on very short-term fasting; therefore, the period of fasting was 2 d. One of the possible explanations for differences in results is the fasting period. The gene expression of adiponectin was higher for fasting than feeding, as in previous studies^28^.

The results of metabolome analysis via NMR were used in subsequent multivariate analysis. OPLS-DA discriminated the feeding from fasting stages (Fig. 3B), and PC1 yielded an adequate, but not perfect, separation of the feeding from fasting stages. The separation factor was thought to be inosine, which was located on the negative side of the P1 matrix, because inosine is an ATP-derived product. The amount of inosine increases with the degradation of muscle cells^50^. Frozen muscle samples were stored for two months, so these results were likely affected by cryogenic preservation. However, a substrate is required for chemical reactions associated with cell degradation. The amount of ATP, the energy-generating purine nucleotide and the source of inosine, is thought to be high during feeding as compared to fasting. On the other hand, leucine and isoleucine appeared to contribute to the positive side of P[1] (Fig. S10A). Leucine and isoleucine are metabolised in the skeletal muscle^51^, and the amounts of these substances tended to increase on the second day of fasting. Moreover, leucine and isoleucine are utilised as an energy source under energy depletion conditions^52, 53^, so these substances are a potential source of energy during fasting.

Integrative analysis of the metabolome and transcriptome data indicated the presence of metabolites and genes related to circadian rhythms and feeding. Regarding circadian rhythms, lactate, malate, and fumarate showed diurnal circadian profiles, and mannose, G6P, F6P, and inosine exhibited nocturnal circadian profiles (Fig. 4). During fasting, the amounts of leucine and isoleucine, which are known as ketogenic amino acids, increased (Fig. 4). These amino acids were contained in fish feed (Fig. S14). Amino acid derived from feed is thought to be utilized during feeding. On the other hand, amino acid derived from muscular protein degradation is thought to be utilized during fasting. A similar phenomenon occurs in fish brains in that ketone bodies increase during fasting^54^. However, the gene expression levels of the branched-chain amino acid (BCAA) metabolic pathway decreased during fasting. Further studies would be needed to clarify the mechanisms. Of the purine metabolism, the dynamics of inosine(HxR) and IMP showed contrary patterns. IMP dehydrogenase 1b gene expression exhibited circadian rhythm, and its expression levels were high in the daytime (Fig. 4). IMP dehydrogenase is the enzyme that converts IMP to XMP, and its expression is known to be displayed in a circadian fashion. Zebrafish embryos expressed three kinds of IMP dehydrogenase; 1a, 1b, and 2, and all showed 24-h rhythms^40^. In leopard coral grouper muscle, IMP dehydrogenase 1a exhibited almost no expression, and IMP dehydrogenase 2 did not show a circadian pattern (Fig. S13). Adenosine kinase and GMP reductase also displayed in a circadian fashion. Those were predicted that the levels of gene expression would increase as the amount of IMP increased. On the other hand, the expression levels of the 5′-nucleotidase, cytosolic IAa gene were high during the night. 5ʹ-nucleotidase is the enzyme that produces inosine from IMP, and six kinds of these genes exist. In mammalian liver, 5′-nucleotidase has been known to exhibit circadian patterns^55^. In this study, only the 5′-nucleotidase, cytosolic IAa gene showed a circadian expression pattern, and the levels of expression increased during feeding as compared to fasting. In the TCA cycle and glycolysis, four kinds of aldolase genes were expressed. Only the aldolase C, fructose-bisphosphate b gene showed a nocturnal expression pattern. The expression pattern of aldolase is known to exhibit a circadian rhythm; however, the peak expression times were different from those in mammalian liver^56^. The amount of malate varied in a circadian fashion and was high at ZT10 in this study. In the muscle, four kinds of malate dehydrogenase were expressed. The dynamics of malate dehydrogenases and their relationship with NADH in muscle have been previously reported^57^. Malate dehydrogenase 2 was the most abundant in the present study. Malate dehydrogenase 1Aa, NAD (soluble) was found to exhibit a circadian pattern, and the expression levels were high at ZT10. This expression pattern corresponded to that of the metabolite. Moreover, the expression levels were low during nutritional depletion. The levels of isocitrate dehydrogenase 3 (NAD+) beta and gamma gene expression were low during fasting. Mammalian isocitrate dehydrogenase has been known to alter its expression based on nutritional conditions^58^.

In the present study, we profiled the gene expression and metabolites associated with food depletion and circadian rhythms in fish muscle tissues. The transcriptomes of 19,737 genes and metabolomes of 407 peaks were analysed. This comprehensive study of the energy production pathway provides novel and fundamental metabolic information for the leopard coral grouper. This information contains not only the metabolic pathways of amino acids and carbohydrates, but also those of hormonal control and its related genes. Currently, excess amount of feed causes the accumulation of fat in the muscle and viscera. Improvement in aquaculture technology is needed with regard to feed efficiency and metabolic control. Our result would help finding out the efficient time and the amount of feeding, and the best composition of feed ingredient. For example, by comparing feed condition with fasted condition, we could know which substances are derived from feed and which substances can be absorbed and utilized. From circadian data, we could know when the best time to intake the specific nutrition is. Our obtained fundamental data would give us hints for the feeding improvements.

## Methods

### Fish and sampling

Hatchery-reared leopard coral groupers (approximately 60 g) were maintained in 60 kL tanks (11.1 kg/m^2^) in a flow-through system at the Yaeyama Laboratory, Seikai National Fisheries Research Institute, Fisheries Research Agency. The water temperature ranged from 21.5 to 22.6 °C, the water pH ranged from 7.2 to 8, the salinity of seawater was 35–37%, and the facility was illuminated by sunlight with a natural photoperiod (11 L:13 D) during the winter. Before experiment, fish were fed twice a day (ZT2 and ZT10) to satiation. The fish were fasted for the first two days of the experiment, and were then fed (ZT2 and ZT10) to satiation for the next two experimental days. The amount of feed was about 1.5% of the body weight. Nutrition facts of fish feed are: protein (more than 46.0%), fat (more than 10.0%), carbohydrate (less than 15.0%), fiber (less than 2.5%), calcium (more than 2.0%), phosphorus (more than 1.0%). The concentration of dissolved oxygen was 6.0–7.5 mg/L. The concentration of ammonia was 0.1 mg/L in fasting period and 0.4–0.5 mg/L after feeding. Three to twelve fish were sampled individually in each time point. Samples were collected every 4 h, with the exception of the evenings of Day 1 and Day 3. Fish were anesthetised with 0.2% 2-phenoxyethanol (Wako, Osaka, Japan) prior to sampling. It took about 10–15 min from anesthetized to frozen. All experiments were conducted according to principles and procedures approved by the guidelines for the care and use of live fish at National Research Institute of Fisheries Science and RIKEN.

### RNA extraction

Muscle tissues were dissected from the fish after anaesthesia was administered, and the samples were immersed and maintained in RNA stabilisation solution (RNA-later, Thermo Fisher Scientific, Waltham, MA, USA). Total RNA was extracted using the RNeasy Lipid Tissue Mini Kit (Qiagen GmbH, Hilden, Germany), according to the manufacturer’s protocol. RNA quality was evaluated based on the proportion of rRNA using an Agilent 2100 Bioanalyzer RNA 6000 Nano Kit (Agilent Technologies, Palo Alto, CA, USA).

### cDNA library construction and sequencing

Total RNA (500 ng) was processed using the SureSelect Strand Specific RNA Library Kit (Agilent). cDNA libraries were constructed with barcodes to enable the multiplexing of pools, and libraries were sequenced using an Illumina Nextseq platform equipped with a 75 bp single-end module.

### Illumina read assembly and transcriptome profiling

Sequencing and multi-index adaptors were trimmed from the sequenced raw reads. Low-quality sequences were then removed and *de novo* assembly performed using CLC Genomics Workbench 8.1. Removal of low quality sequence parameter (limit) was 0.05, and removal of ambiguous nucleotides was 2 (maximal 2 nucleotides allowed). The generated contigs were analysed using the BLAST program, which compared the sequences to the zebrafish Ensembl protein database (http://www.ensembl.org/). Contigs were sorted by corresponding Ensembl IDs, and representative sequences were selected based on the highest e-value, because the same Ensembl ID was associated with more than one contig. The selected sequences were used as references for the read mapping analysis that was performed using CLC Genomics Workbench 8.1, and the results were expressed as reads per kilobase of exon model per million mapped reads (RPKM)^59^. Quantitative PCR validation by selected genes from the Figure S5 was performed. Gene ontology annotation and analyses were conducted using Panther software (http://www.pantherdb.org/).

### Quantitative PCR

Genomic DNA was eliminated from total RNA by TURBO DNA-free™ Kit (Thermo Fisher Scientific, Waltham, MA). First-strand cDNA was synthesized from 1 μg of total RNA using the SuperScript™ III First-Strand Synthesis System (Thermo Fisher Scientific) with random hexamers. The PCR reaction mixture consisted of each primer pair (0.2 μM) and GoTaq® qPCR Master Mix (Promega, Madison, WI). Primer sequences and annealing temperature are shown in Supplemental Table S5. Data were analyzed using the Thermal Cycler Dice® TP800 (Takara, Shiga, Japan) and Thermal Cycler Dice® Real Time System Software Ver.5.11. Dissociation curve analysis was performed to ensure only a single peak was amplified. The expression levels of mRNA were normalized to EF1α in respective samples. Data were expressed Z-score and means ± SEM.

### Statistics

Statistical analyses were conducted using the DGE test, and differences at *p* < 0.05 were considered significant. All analyses were conducted using CLC Genomics Workbench 8.1. Transcriptomic data were analysed by a principal component analysis (PCA) using R software. The data were visualized in the form of the PCA score plots. For qPCR, Statistical differences were determined by one‐way analysis of variance (ANOVA), followed by the Tukey’s test. Differences at P < 0.05 were considered significant. All analyses were performed by a computer program, Prism (GraphPad Software, San Diego, CA).

### NMR spectroscopy

Tissue samples were stored at −80 °C immediately after dissection. Sample were then shipped and stored at −20°C until NMR measurement. Samples were soaked into liquid nitrogen followed by lyophilized for sample preparation. Subsequently sample preparation for high-resolution^1^H NMR spectroscopy was conducted as previously described^13–15^. Briefly, 600 μL of D_2_O/KPi (100 mM, pH 7.0) was added to 18 mg of milled muscle tissue samples, and metabolite mixtures were extracted via shaking at 338 K for 15 min. The high-resolution^1^H NMR spectra were recorded with an AVANCE II-700 Spectrometer (Bruker, Billerica, MA, USA) equipped with an inverse triple-resonance cryogenic probe with a Z-axis gradient for 5-mm sample diameters that operated at 700.03 MHz for^1^H. All NMR samples were maintained at 298 K. The chemical shifts were referenced based on the methyl groups of 4,4-dimethyl-4-silapentane-1-sulfonic acid (DSS, 0.00/0.00 ppm [δ_13C_/δ_1H_]). 1D-^1^H, 2D-Jres,^1^H-^13^C-HSQC, and^1^H-^13^C-HSQC-TOCSY experiments were employed for signal assignment. The annotations and assignments of metabolites detected in the NMR spectra analysis were performed using the SpinAssign program found on the PRIMe website (http://prime.psc.riken.jp/)^60^, the web-based SpinCouple tool (http://emar.riken.jp/spincpl)^61^, and the Human Metabolome Database (http://www.hmdb.ca/)^62^.

### Data processing and analysis

2D-Jres NMR spectra were processed by TopSpin software (Bruker Biospin, Rheinstetten, Germany). Tilt correction and symmetrisation was performed and the projection of the 2D spectrum obtained. Next, 407 regions of interest (ROI) were manually defined using Revolution R Open software^63–65^. Projection spectra in ROI were then converted to numeric data and normalised. Metabolomic data was analysed by a projection to a latent structure-discriminant analysis (PLS-DA) and orthogonal PLS-DA (OPLS-DA) using R software as previously described^66–68^. The data were visualised as score plots and loading plots. Correlation test were performed with R software. Positive and negative correlated gene and metabolites with four teacher pattern were found. Four kinds of teacher datasets were: high in the daytime and night-time (pattern 1), low in the fasting (pattern 2), gradually decrease and increase (pattern 3), acute responding to feeding (pattern 4) (Fig. S2).

## Electronic supplementary material

Supplmentary figures S1-S14.

Table S1

Table S2A

Table S2B

Table S2C

Table S3A

Table S3B

Table S3C

Table S3D

Table S3E

Table S3F

Table S4

Table S5

## References

[1] Tilman D, Clark M (2014). Global diets link environmental sustainability and human health. Nature.

[2] Xie J (2011). Ecological mechanisms underlying the sustainability of the agricultural heritage rice–fish coculture system. Proc Natl Acad Sci USA.

[3] Halpern BS (2012). An index to assess the health and benefits of the global ocean. Nature.

[4] Sumaila UR (2015). Winners and losers in a world where the high seas is closed to fishing. Sci rep.

[5] Pauly D, Zeller D (2016). Catch reconstructions reveal that global marine fisheries catches are higher than reported and declining. Nat commun.

[6] Cheung WW (2013). Shrinking of fishes exacerbates impacts of global ocean changes on marine ecosystems. Nat Clim Change.

[7] Mathiesen, A. The State of the World Fisheries and Aquaculture 2012 (2012).

[8] Ogata Y (2012). ECOMICS: a web-based toolkit for investigating the biomolecular web in ecosystems using a trans-omics approach. PLoS One.

[9] Tian C (2007). Top-down phenomics of Arabidopsis thaliana metabolic profiling by one-and two-dimensional nuclear magnetic resonance spectroscopy and transcriptome analysis of albino mutants. J Biol Chem.

[10] Samuelsson LM, Larsson DG (2008). Contributions from metabolomics to fish research. Mol Biosyst.

[11] Samuelsson LM, Bjorlenius B, Forlin L, Larsson DG (2011). Reproducible (1)H NMR-based metabolomic responses in fish exposed to different sewage effluents in two separate studies. Environ Sci Technol.

[12] Wagner L, Trattner S, Pickova J, Gómez-Requeni P, Moazzami AA (2014). 1 H NMR-based metabolomics studies on the effect of sesamin in Atlantic salmon (Salmo salar). Food Chem.

[13] Yoshida S, Date Y, Akama M, Kikuchi J (2014). Comparative metabolomic and ionomic approach for abundant fishes in estuarine environments of Japan. Sci Rep.

[14] Asakura T, Sakata K, Yoshida S, Date Y, Kikuchi J (2014). Noninvasive analysis of metabolic changes following nutrient input into diverse fish species, as investigated by metabolic and microbial profiling approaches. PeerJ.

[15] Misawa T, Wei F, Kikuchi J (2016). Application of Two-Dimensional Nuclear Magnetic Resonance for Signal Enhancement by Spectral Integration Using a Large Data Set of Metabolic Mixtures. Anal Chem.

[16] Ogura T, Hoshino R, Date Y, Kikuchi J (2016). Visualization of Microfloral Metabolism for Marine Waste Recycling. Metabolites.

[17] Fabinyi M (2012). Historical, cultural and social perspectives on luxury seafood consumption in China. Environ Conserv.

[18] Moon TW, Johnston IA (1980). Starvation and the activities of glycolytic and gluconeogenic enzymes in skeletal muscles and liver of the plaice,Pleuronectes platessa. J Comp Physiol.

[19] Vatine G, Vallone D, Gothilf Y, Foulkes NS (2011). It’s time to swim! Zebrafish and the circadian clock. FEBS Lett.

[20] Vakili H, Jin Y, Cattini PA (2016). Evidence for a circadian effect on the reduction of human growth hormone gene expression in response to excess caloric intake. J Biol Chem.

[21] Johnston IA, Sidell BD, Driedzic WR (1985). Force-velocity characteristics and metabolism of carp muscle fibres following temperature acclimation. J ExpBiol.

[22] Cates MS, Teodoro ML, Phillips GN (2002). Molecular mechanisms of calcium and magnesium binding to parvalbumin. Biophys J.

[23] Dominguez, R. & Holmes, K. C. In *Annual Review of Biophysics, Vol 40* Vol. 40 *Annual Review of Biophysics* (eds D. C. Rees, K. A. Dill, & J. R. Williamson) 169–186 (Annual Reviews, 2011).

[24] Palstra AP (2013). Deep RNA sequencing of the skeletal muscle transcriptome in swimming fish. PLoS One.

[25] Shibata M (2016). Transcriptomic features associated with energy production in the muscles of Pacific bluefin tuna and Pacific cod. Biosci Biotech Biochem.

[26] Garcia de la serrana D, Estévez A, Andree K, Johnston IA (2012). Fast skeletal muscle transcriptome of the Gilthead sea bream (Sparus aurata) determined by next generation sequencing. BMC Genomics.

[27] Calduch-Giner JA (2013). Deep sequencing for de novo construction of a marine fish (Sparus aurata)transcriptome database with a large coverage of protein-coding transcripts. BMC Genomics.

[28] Kondo H (2012). Effects of feed restriction on the expression profiles of the glucose and fatty acid metabolism-related genes in rainbow trout Oncorhynchus mykiss muscle. Fish Sci.

[29] Rimoldi S, Benedito-Palos L, Terova G, Pérez-Sánchez J (2016). Wide-targeted gene expression infers tissue-specific molecular signatures of lipid metabolism in fed and fasted fish. Rev Fish Biol Fisheries.

[30] Chatterjee S, Ma K (2016). Circadian clock regulation of skeletal muscle growth and repair. F1000Research.

[31] Tamai TK, Young LC, Whitmore D (2007). Light signaling to the zebrafish circadian clock by Cryptochrome 1a. Proc Natl Acad Sci USA.

[32] Amaral IP, Johnston IA (2012). Circadian expression of clock and putative clock-controlled genes in skeletal muscle of the zebrafish. Am J Physiol Regul Integr Comp Physiol.

[33] Buckingham M (1992). Making muscle in mammals. Trends Genet.

[34] Andrews JL (2010). CLOCK and BMAL1 regulate MyoD and are necessary for maintenance of skeletal muscle phenotype and function. Proc Natl Acad Sci.

[35] Björnsson BT (2002). Growth Hormone Endocrinology of Salmonids: Regulatory Mechanisms and Mode of Action. Fish Physiol Biochem.

[36] Mullur R, Liu Y-Y, Brent GA (2014). Thyroid hormone regulation of metabolism. Physiol Rev.

[37] Yen PM (2001). Physiological and molecular basis of thyroid hormone action. Physiol Rev.

[38] Yamauchi T (2003). Cloning of adiponectin receptors that mediate antidiabetic metabolic effects. Nature.

[39] Gómez-Abellán P (2010). Circadian Expression of Adiponectin and Its Receptors in Human Adipose Tissue. Endocrinology.

[40] Li Y (2015). Integrative Analysis of Circadian Transcriptome and Metabolic Network Reveals the Role of De Novo Purine Synthesis in Circadian Control of Cell Cycle. Plos Comput Biol.

[41] Lowenstein JM (1972). Ammonia production in muscle and other tissues: the purine nucleotide cycle. Physiol Rev.

[42] Gumbmann M, Tappel A (1962). The tricarboxylic acid cycle in fish. Arch Biochem Biophys.

[43] Knox D, Walton M, Cowey C (1980). Distribution of enzymes of glycolysis and gluconeogenesis in fish tissues. Mar Biol.

[44] Fujisawa K (2016). Circadian variations in the liver metabolites of medaka (Oryzias latipes). Sci Rep.

[45] Hirota T (2002). Glucose down-regulates Per1 and Per2 mRNA levels and induces circadian gene expression in cultured Rat-1 fibroblasts. J Biol Chem.

[46] Enes P, Panserat S, Kaushik S, Oliva-Teles A (2009). Nutritional regulation of hepatic glucose metabolism in fish. Fish Physiol Biochem.

[47] Suarez RK, Mommsen TP (1987). Gluconeogenesis in teleost fishes. Can J Zool Rev Can Zool.

[48] Garcia de la serrana D, Estévez A, Andree K, Johnston IA (2012). Fast skeletal muscle transcriptome of the Gilthead sea bream (Sparus aurata) determined by next generation sequencing. BMC Genomics.

[49] Bower NI, Taylor RG, Johnston IA (2009). Phasing of muscle gene expression with fasting-induced recovery growth in Atlantic salmon. Front Zool.

[50] Howgate P (2006). A review of the kinetics of degradation of inosine monophosphate in some species of fish during chilled storage. Int J Food Sci Technol.

[51] van den Thillart G (1986). Energy metabolism of swimming trout (Salmo gairdneri). J Comp Physiol B.

[52] Gillis T, Ballantyne J (1996). The effects of starvation on plasma free amino acid and glucose concentrations in lake sturgeon. J Fish Biol.

[53] Holecek M, Sprongl L, Tilser I (2001). Metabolism of branched-chain amino acids in starved rats: the role of hepatic tissue. Physiol Res.

[54] Soengas JL, Strong EF, Andres MD (1998). Glucose, Lactate, and b‐Hydroxybutyrate Utilization by Rainbow Trout Brain: Changes during Food Deprivation. Physiol Biochem Zool.

[55] Von Mayersbach H, Klaushofer K (1979). Circadian variations of 5′-nucleotidase activity in rat liver. Cell Mol Biol Incl Cyto Enzymol.

[56] Reddy AB (2006). Circadian Orchestration of the Hepatic Proteome. Curr Biol.

[57] Fahien LA (1999). Ability of Cytosolic Malate Dehydrogenase and Lactate Dehydrogenase to Increase the Ratio of NADPH to NADH Oxidation by Cytosolic Glycerol-3-phosphate Dehydrogenase. Arch Biochem Biophys.

[58] Koh H-J (2004). Cytosolic NADP+ -dependent isocitrate dehydrogenase plays a key role in lipid metabolism. J Biol Chem.

[59] Mortazavi A, Williams BA, McCue K, Schaeffer L, Wold B (2008). Mapping and quantifying mammalian transcriptomes by RNA-Seq. Nat Meth.

[60] Chikayama E (2010). Statistical indices for simultaneous large-scale metabolite detections for a single NMR spectrum. Anal Chem.

[61] Kikuchi J (2016). SpinCouple: Development of a Web Tool for Analyzing Metabolite Mixtures via Two-Dimensional J-Resolved NMR Database. Anal Chem.

[62] Wishart DS (2013). HMDB 3.0–The Human Metabolome Database in 2013. Nucleic Acids Res.

[63] Vergara F, Kikuchi J, Breuer C (2016). Artificial Autopolyploidization Modifies the Tricarboxylic Acid Cycle and GABA Shunt in Arabidopsis thaliana Col-0. Sci Rep.

[64] Shiokawa Y, Misawa T, Date Y, Kikuchi J (2016). Application of Market Basket Analysis for the Visualization of Transaction Data Based on Human Lifestyle and Spectroscopic Measurements. Anal Chem.

[65] Lewis IA, Schommer SC, Markley JL (2009). rNMR: open source software for identifying and quantifying metabolites in NMR spectra. Magn Reson Chem.

[66] Asakura T, Date Y, Kikuchi J (2014). Comparative analysis of chemical and microbial profiles in estuarine sediments sampled from Kanto and Tohoku regions in Japan. Anal Chem.

[67] Ito K, Sakata K, Date Y, Kikuchi J (2014). Integrated analysis of seaweed components during seasonal fluctuation by data mining across heterogeneous chemical measurements with network visualization. Anal Chem.

[68] Motegi H (2015). Identification of Reliable Components in Multivariate Curve Resolution-Alternating Least Squares (MCR-ALS): a Data-Driven Approach across Metabolic Processes. Sci Rep.

