# Supplementary material for: Trans-omics approaches used to characterise fish nutritional biorhythms in leopard coral grouper (Plectropomus leopardus)
Source: Sci Rep. 2017 Aug 24;7:9372. doi: 10.1038/s41598-017-09531-4 (PMC5570933; doi:10.1038/s41598-017-09531-4)
Supplement: Supplementary file 1 — Supplmentary figures S1-S14. [file 41598_2017_9531_MOESM1_ESM.pdf]

Trans-omics approaches used to characterise fish nutritional biorhythms in leopard coral grouper (*Plectropomus leopardus*)

Miyuki Mekuchi[1,2], Kenji Sakata[1], Tomofumi Yamaguchi[3], Masahiko Koiso[3], and Jun Kikuchi[1,4,5,*]

[1]RIKEN Center for Sustainable Resource Science, 1-7-22 Suehiro-cho, Tsurumi-ku, Yokohama, Kanagawa 230-0045, Japan

[2]National Fishery Research Institute of Fishery Sciences, Fishery Research and Education Organization, 2-12-4, Fukuura, Kanazawa-ku, Yokohama 230-0045, Japan

[3]Research Center for Subtropical Fisheries, 148 Fukaiota, Ishikagi 907-0451 Japan

[4]Graduate School of Medical Life Science, Yokohama City University, 1-7-29 Suehirocho, Tsurumi-ku, Yokohama, Kanagawa 230-0045, Japan

[5]Graduate School of Bioagricultural Sciences, Nagoya University, 1 Furo-cho, Chikusa-ku, Nagoya, Aichi 464-0810, Japan

*Corresponding author: Jun Kikuchi (jun.kikuchi@riken.jp)

Keywords: aquaculture, fishery, gene expression profile, metabolic profile, nuclear magnetic resonance

Supplemental figures

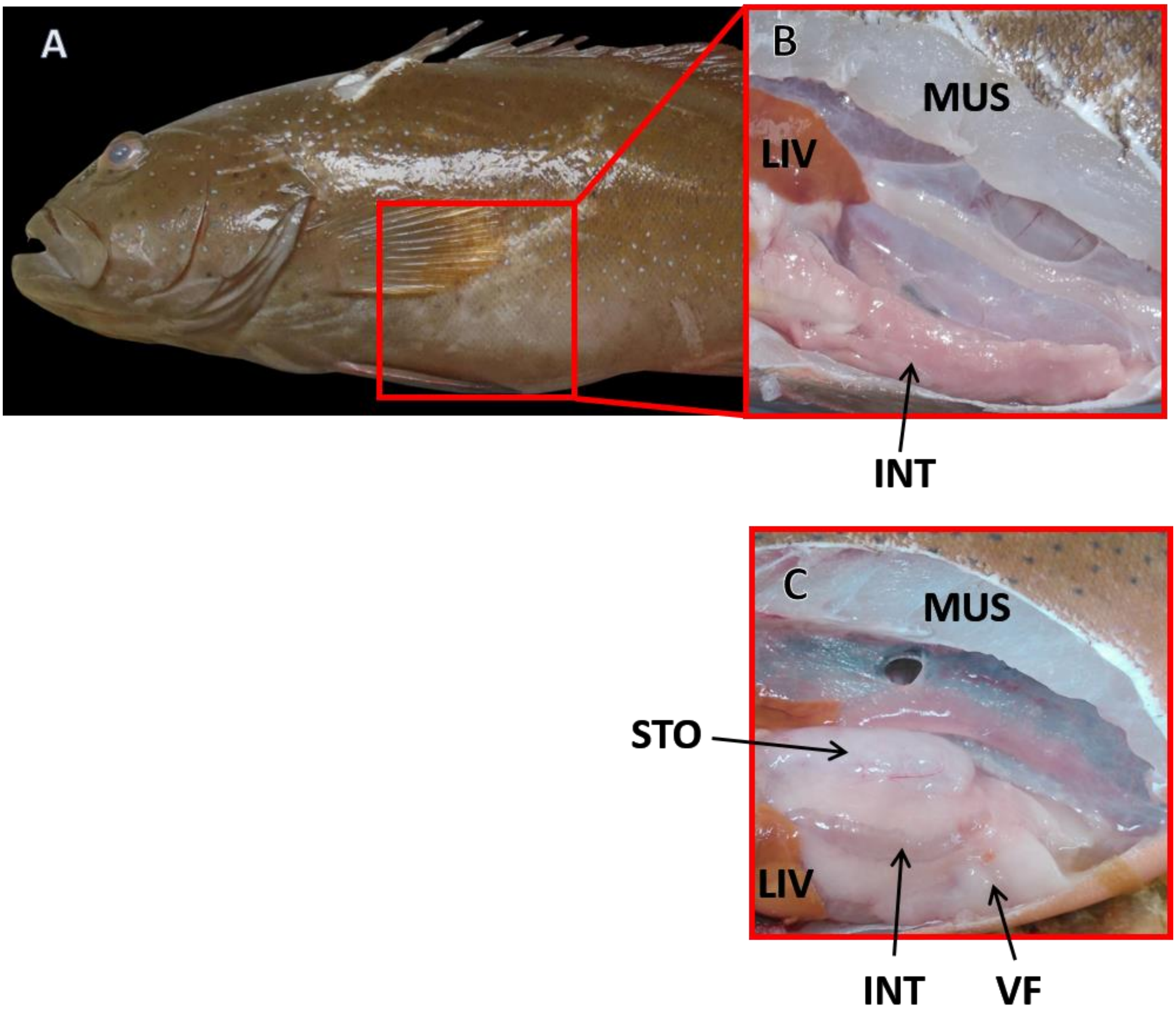

Fig. S1 Anatomy picture of leopard coral grouper.
(A) A picture shows the fish before dissection. (B) An enlargement anatomy picture of wild leopard coral grouper. Liver (LIV), muscle (MUS), intestine (INT), stomach (STO), and visceral fat (VF). (C) An enlargement anatomy picture of farmed leopard coral grouper. The color of wild grouper muscle is transparent, and farmed grouper is whiter. Photographs were taken by Tomofumi Yamaguchi.

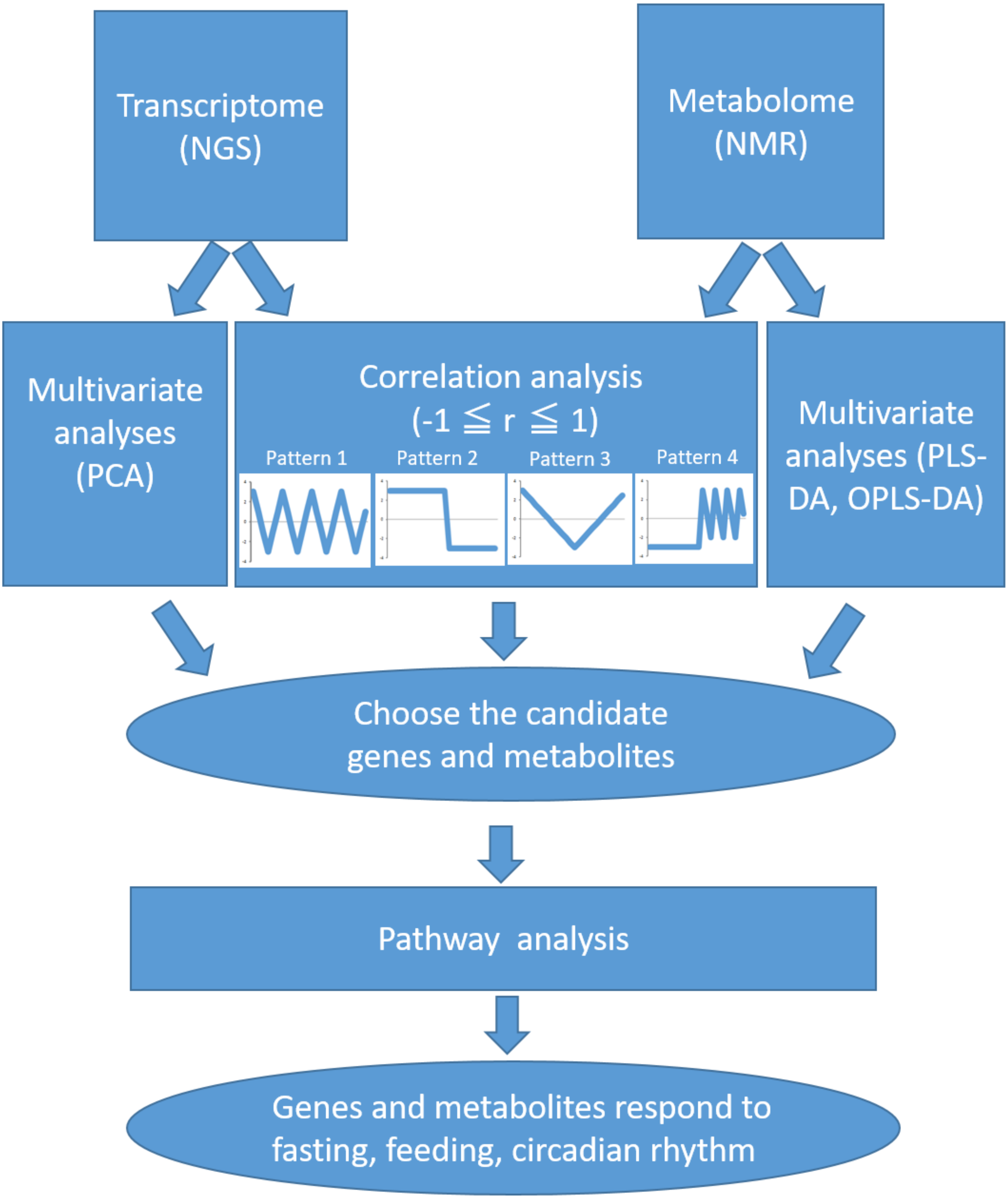

Fig.S2 The schematic diagram of analysis strategies.

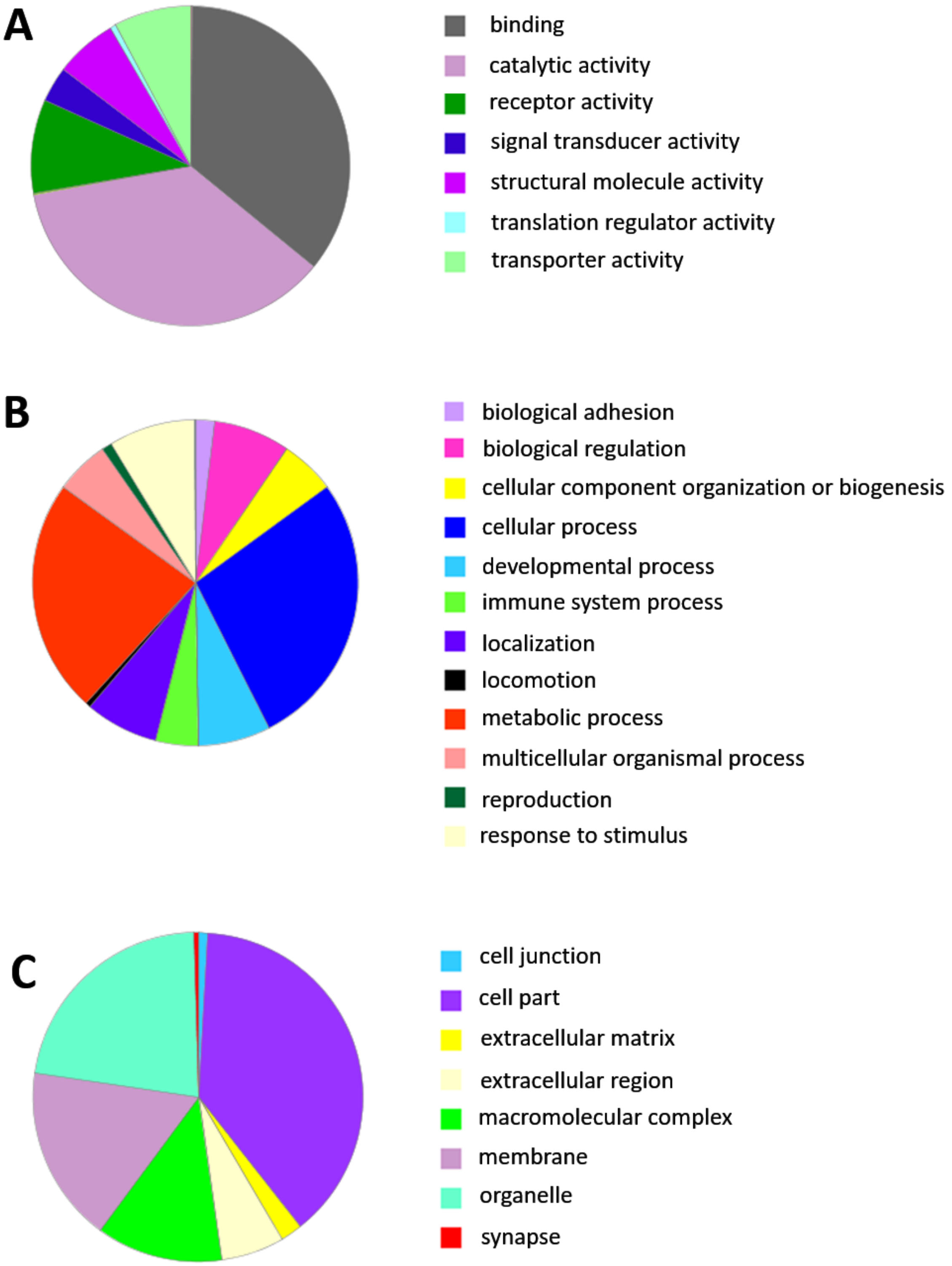

Fig. S3 Gene ontology analysis of leopard coral grouper muscle.
(A) molecular function, (B) biological process, (C) cellular component.

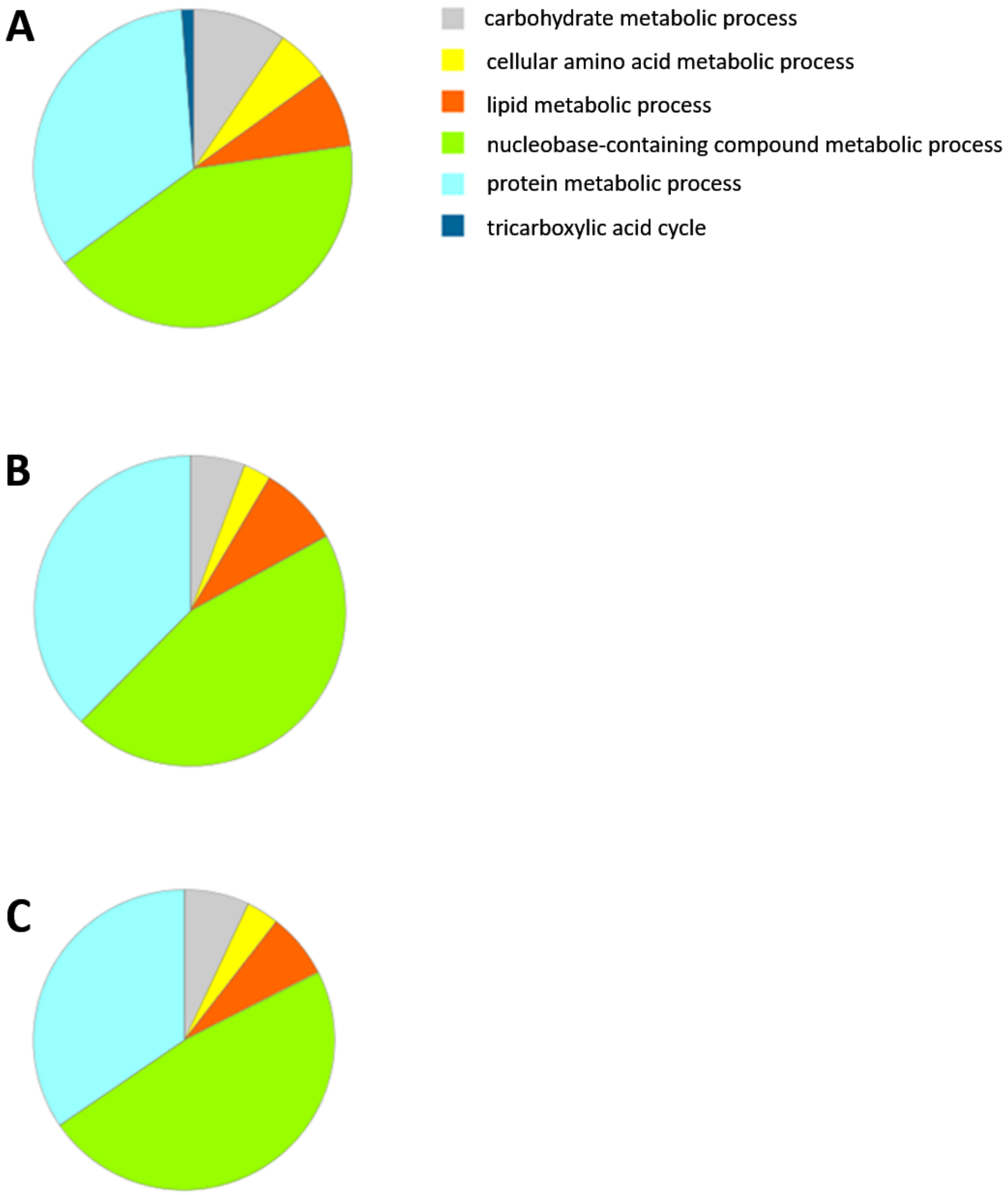

Fig. S4 Gene ontology analysis of three clustered genes.
Three clustered genes were selected from hierarchical clustering analysis (Fig 2A). This analysis was performed focusing on primary metabolism in biological process. (A) cluster 1, the levels of expression were low in fasting (B) cluster 2, the levels of gene expression were high in early fasting (C) cluster 3,the levels of gene expression were high in late fasting.

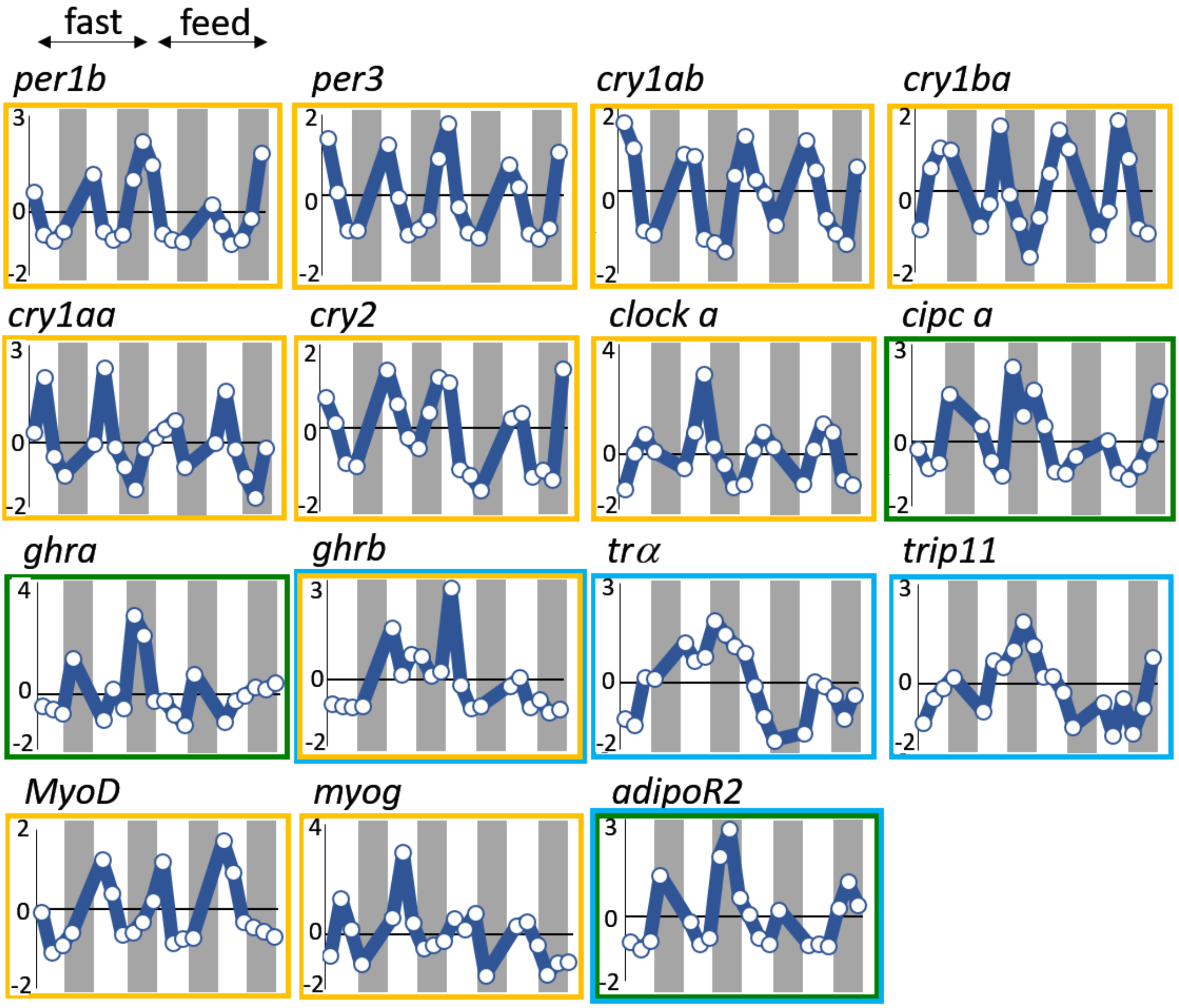

Fig. S5 Gene expression of circadian core genes and hormons.

Period circadian clock 1b (*per1b*),period circadian clock 3 (*per3*), cryptochrome circadian clock 1aa (*cry1aa*), cryptochrome circadian clock 1ab (*cry1ab*), cryptochrome circadian clock 1ba (*cry1ba*), cryptochrome circadian clock 2 (*cry2*), clock circadian regulator a (*clock a*),CLOCK-interacting pacemaker a (*cipc a*), growth hormone receptor a (*ghra*), growth hormone receptor b (*ghrb*), thyroid hormone receptor alpha a (*tra*), thyroid hormone receptor interactor 11 (*trp11*), myogenic differentiation 1 (*myoD*), myogenin (*myog*), and adiponectin receptor 2 (*adipoR2*). Yellow colored gene indicated circadian fashion and expressed high in the day and morning. Green showed nocturnal fashion. Blue showed the upregulation by fasting. Dark colored areas in graphs represent night.

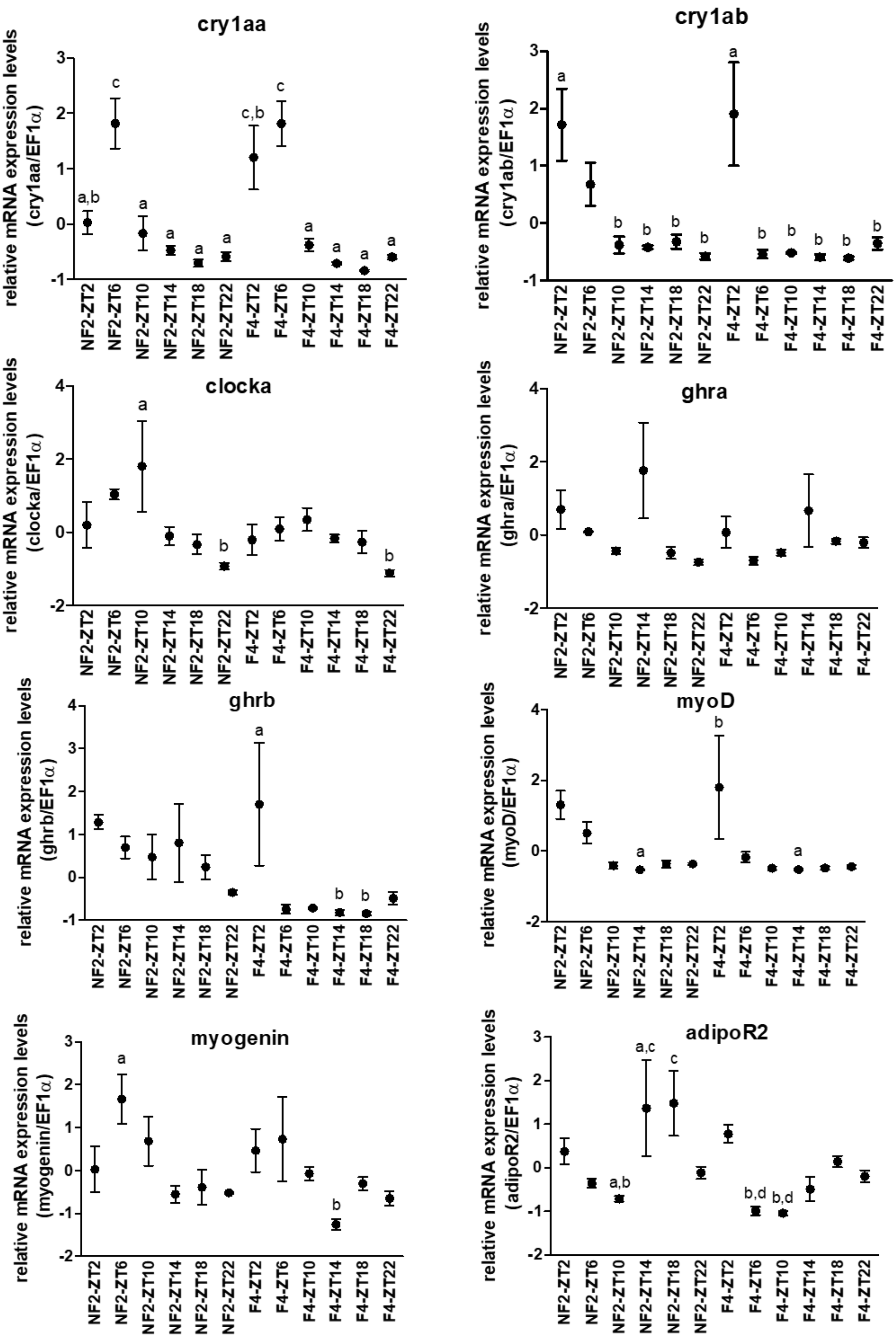

Fig.S6 Quanititative PCR for the validation.

Expression levels of circadian clock 1aa (*cry1aa,* Accession No. LC278355),

cryptochrome circadian clock 1ab (*cry1ab,* LC278367), clock circadian regulator a (*clock a,* LC278356), growth hormone receptor a (*ghra,* LC278357), growth hormone receptor b (*ghrb*, LC278358), myogenic differentiation 1 (*myoD,* LC278359), myogenin (*myog,* LC278360), and adiponectin receptor 2 (*adipoR2,* LC278361) were measured by quantitative real-time PCR. Elongation factor 1α(*EF1α*, LC278366). Second day without feeding (NF2) and fourth day with feeding (F4). ZT stands for Zeitgeber time. Data were expressed as the Z score and mean±SEM. Different letters indicate significant differences at $p < 0.05$.

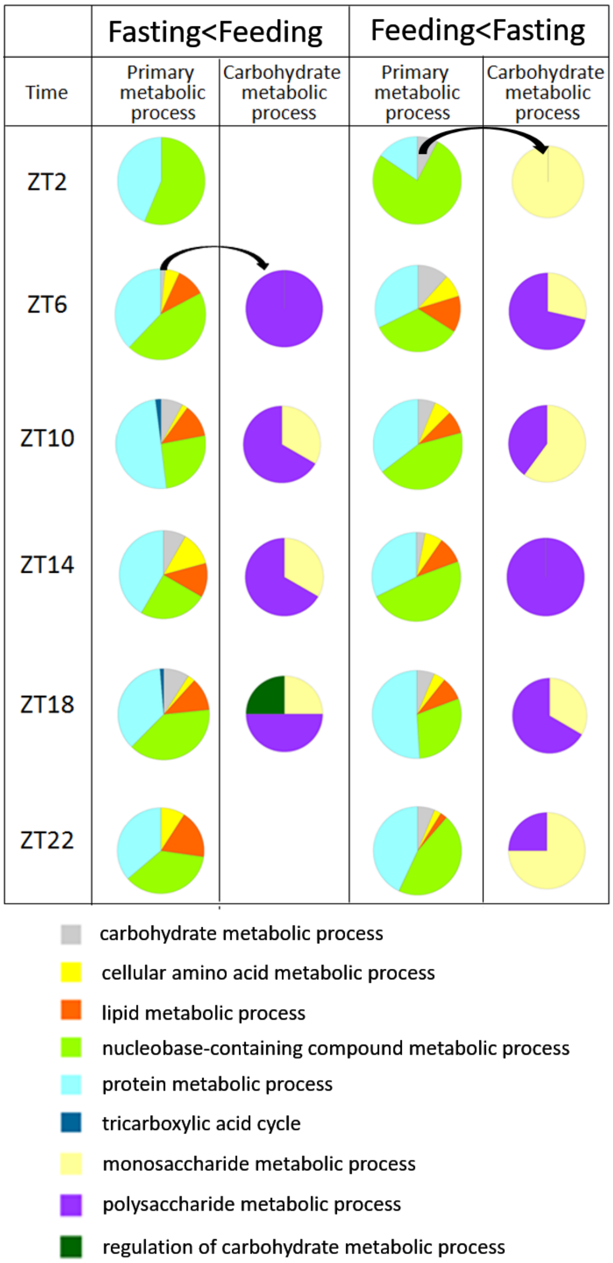

Fig. S7 Gene expression comparisons between fasting and feeding stages. Gene ontology analysis was performed. Analysis was performed using 2 categories data set; upregulated and downregulated genes in fasting compared to feeding. Data set was selected by fold change (FC > 2) and a statistic test ($p < 0.05$). This analysis was focused on a primary metabolic process in the categories of biological process. Subsequent analysis was performed in a lower level of hierarchy, carbohydrate metabolic process.

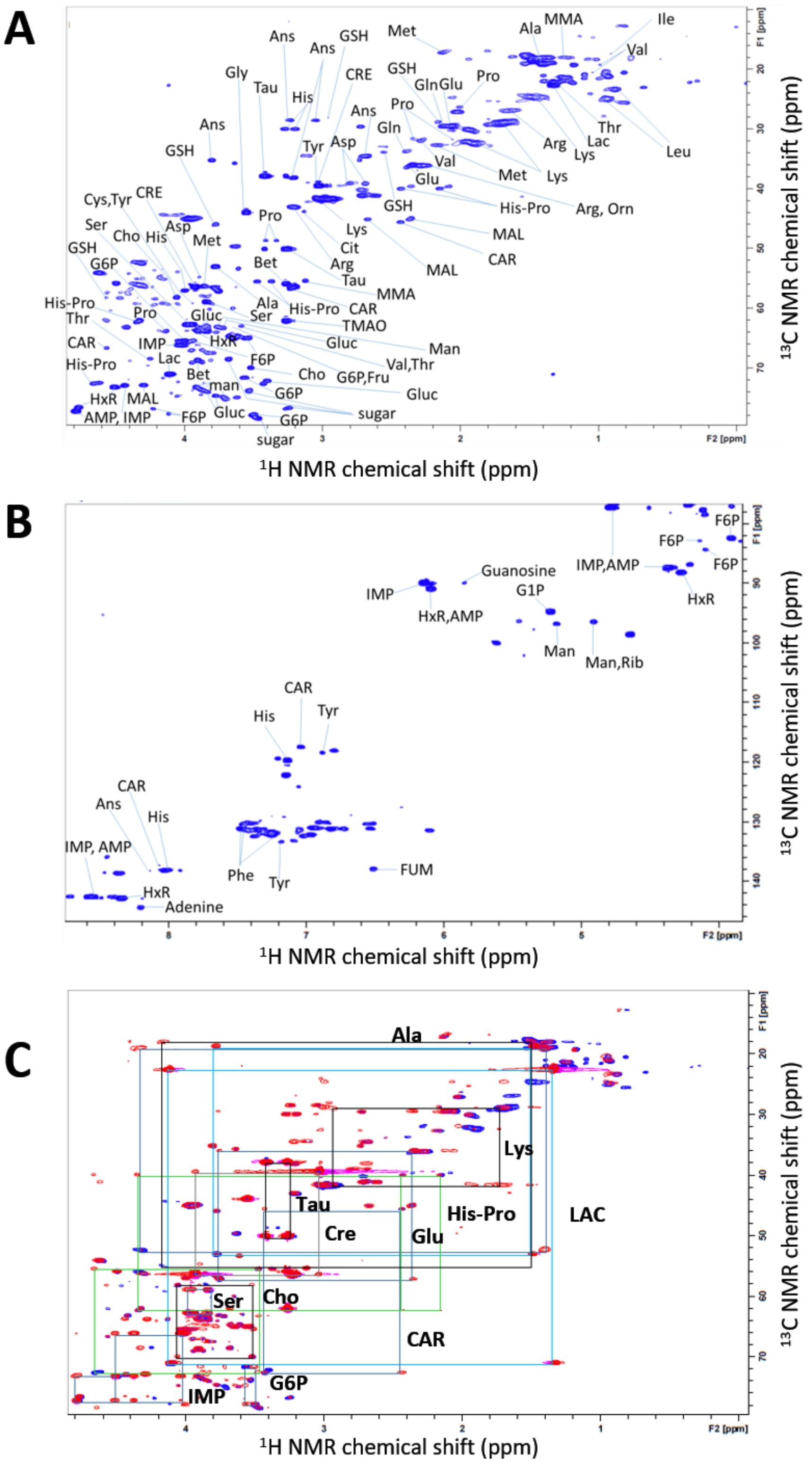

Fig. S8 NMR spectra with metbolite annotations

(A) ^{1}H-^{13}C heteronuclear single quantum coherence (HSQC) NMR spectra of grouper muscle.

Proton range (0 - 4.8 ppm), carbon range (0 - 80 ppm). Annotated substances were filled out. Metabolites and spectra were listed in Table S4. (B) HSQC NMR spectra. Proton range (4 - 8.8 ppm), carbon range (80 - 145 ppm). (C) ^{1}H-^{13}C HSQC- total correlation spectroscopy (TOCSY) NMR spectra. Correlated substances were lined. Abbreviations of substances name were shown in Table S1.

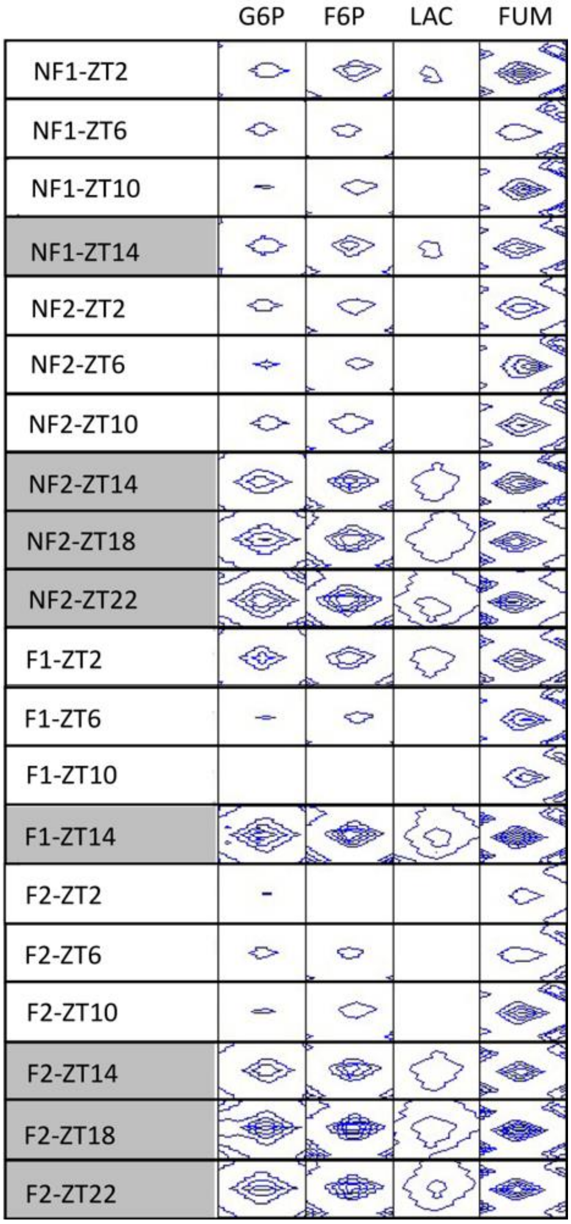

Fig. S9 Expansion of stacked plots of 2D-*J* signals

Stacked plots of two-dimensional (2-D) ^{1}H *J*-resolved spectroscopy spectra. Spectra of interest substances were expanded. Glucose-6-phosphate(G6P), fructose-6-phosphate(F6P), mannose(MAN), and fumarate(FUM).

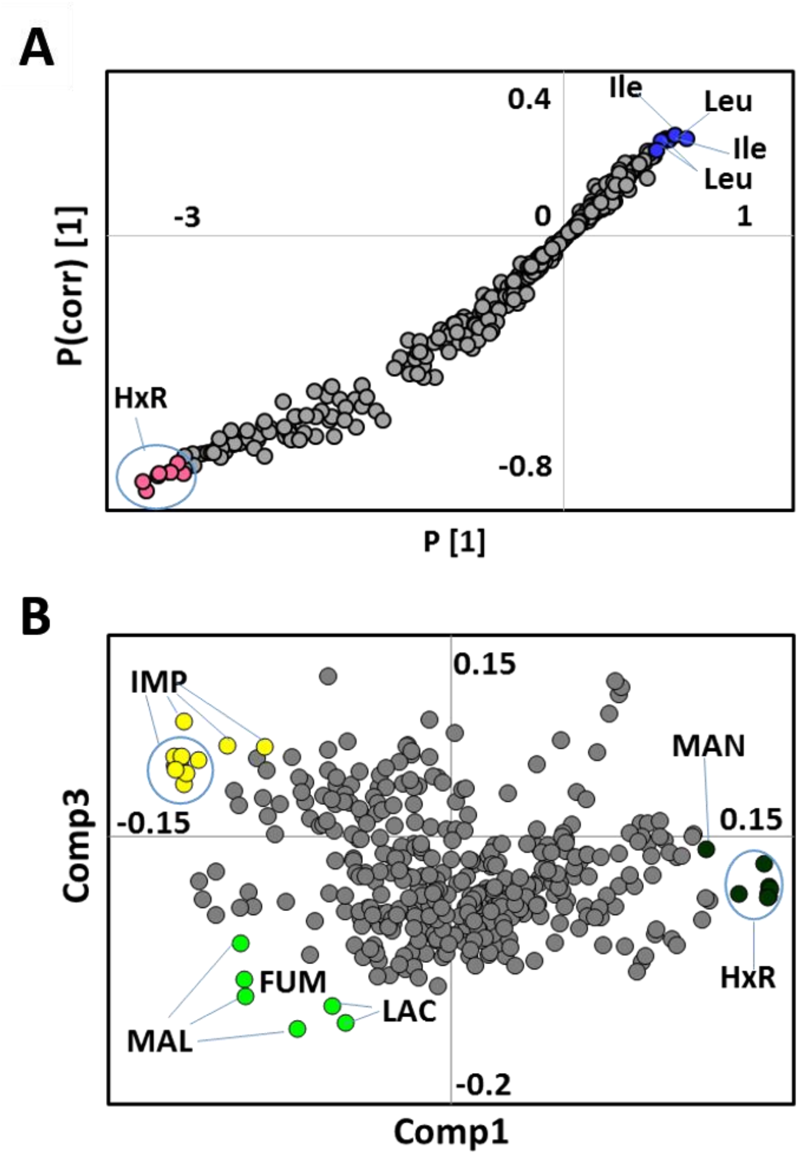

Fig. S10 Loading plot analysis of discrimination analysis.

(A) Discrimination analysis by OPLS-DA. Results were illustrated by a s-plot. The most contributed substances were shown. (B) Discrimination analysis by PLS-DA. Results were illustrated by a scatter plot. The most contributed substances were shown in the figure. Leucine (Leu), isoleucine (Ile), inosine (HxR), hypoxanthine (Hx), inosine monophosphate (IMP), mannose(MAN), and fumarate(FUM), lactate(LAC).

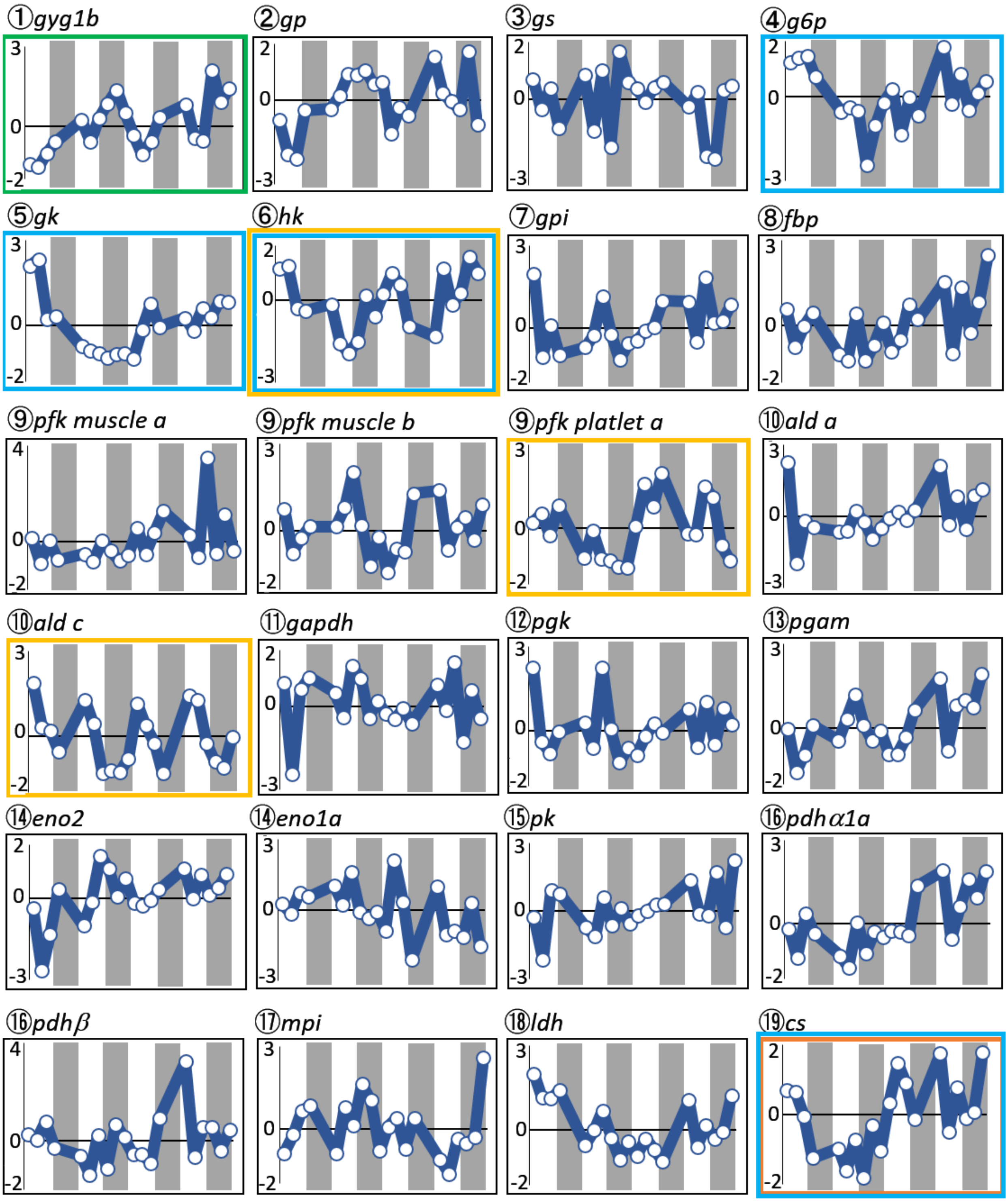

Fig. S11 The dynamics of gene expression on glycolyisis and TCA cycle. Yellow and green colored gene exhibiting dinal and nocturnal fashion, respectively. Blue and pink showed the upregulation by fasting and feeding, respectively. Dark

colored areas represent night. Encircled numbers were corresponded to figure 4. Glycogenin synthase (*gyg1b*), glycogen phosphorylase (*gp*), glycogen synthase (*gs*), glucose-6-phosphate dehydrogenase (*g6p*), glucokinase (*gk*), hexokinase (*hk*), glucose-6-phosphate isomerase (*gpi*), fructose bisphosphatase (*fbp*), phosphofructokinase (*pfk*), aldolase (*ald*), glyceraldehyde 3-phosphate dehydrogenase (*gapdh*), phosphoglycerate kinase (*pgk*), phosphoglycerate mutase (*pgam*), enolase(*eno*), pyruvate kinase (*pk*), pyruvate dehydrogenase(*pdh*), mannose phosphate isomerase (*mpi*),lactate dehydrogenase (*ldh*),citrate synthase (*cs*).

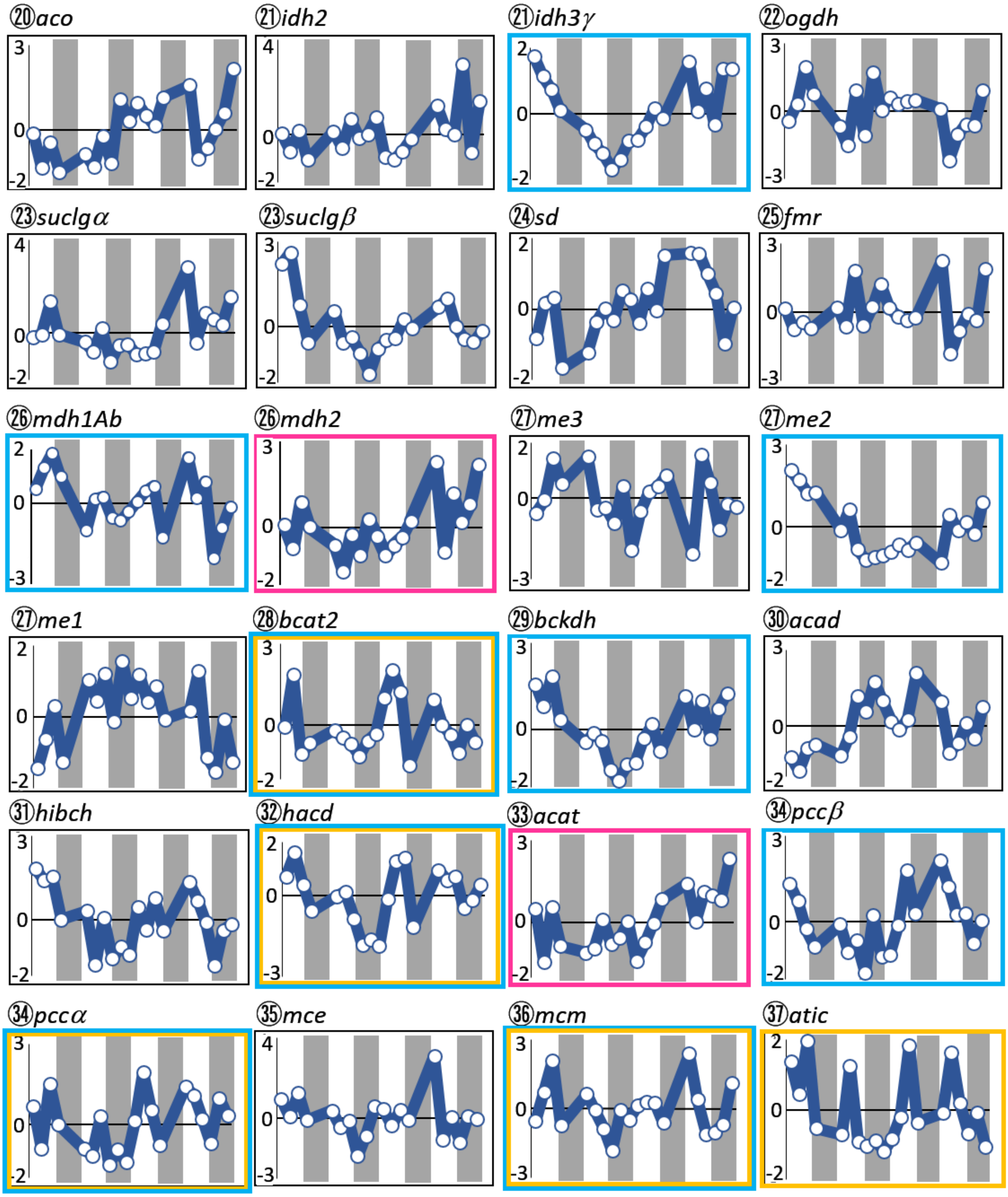

Fig. S12 The dynamics of gene expression on TCA cycle and BCAA metabolism. Yellow and green colored gene exhibiting dinal and nocturnal fashion, respectively. Blue and pink showed the upregulation by fasting and feeding, respectively. Dark

colored areas represent night. Encircled numbers were corresponded to figure 4. Aconitase (*aco*), isocitrate dehydrogenase (*idh*), oxoglutarate dehydrogenase (*ogdh*), succinyl-CoA ligase (*suclg*), succinate dehydrogenase (*sd*), fumarase (*fmr*), malate dehydrogenase (*mdh*), malic enzymes (*me*), branched chain aminotransferase (*bcat*), branched chain keto acid dehydrogenase (*bckdh*), acyl-CoA dehydrogenase (*acad*), 3-hydroxyisobutyryl-CoA hydrolase (*hibch*), 3-hydroxyacyl dehydrogenase (*hacd*), acetyl-CoA acetyltransferase (*acat*), propionyl-CoA carboxylase (*pcc*), methylmalonyl-CoA epimerase (*mce*), methylmalonyl-CoA mutase (*mcm*), IMP cyclohydrase (*atic*).

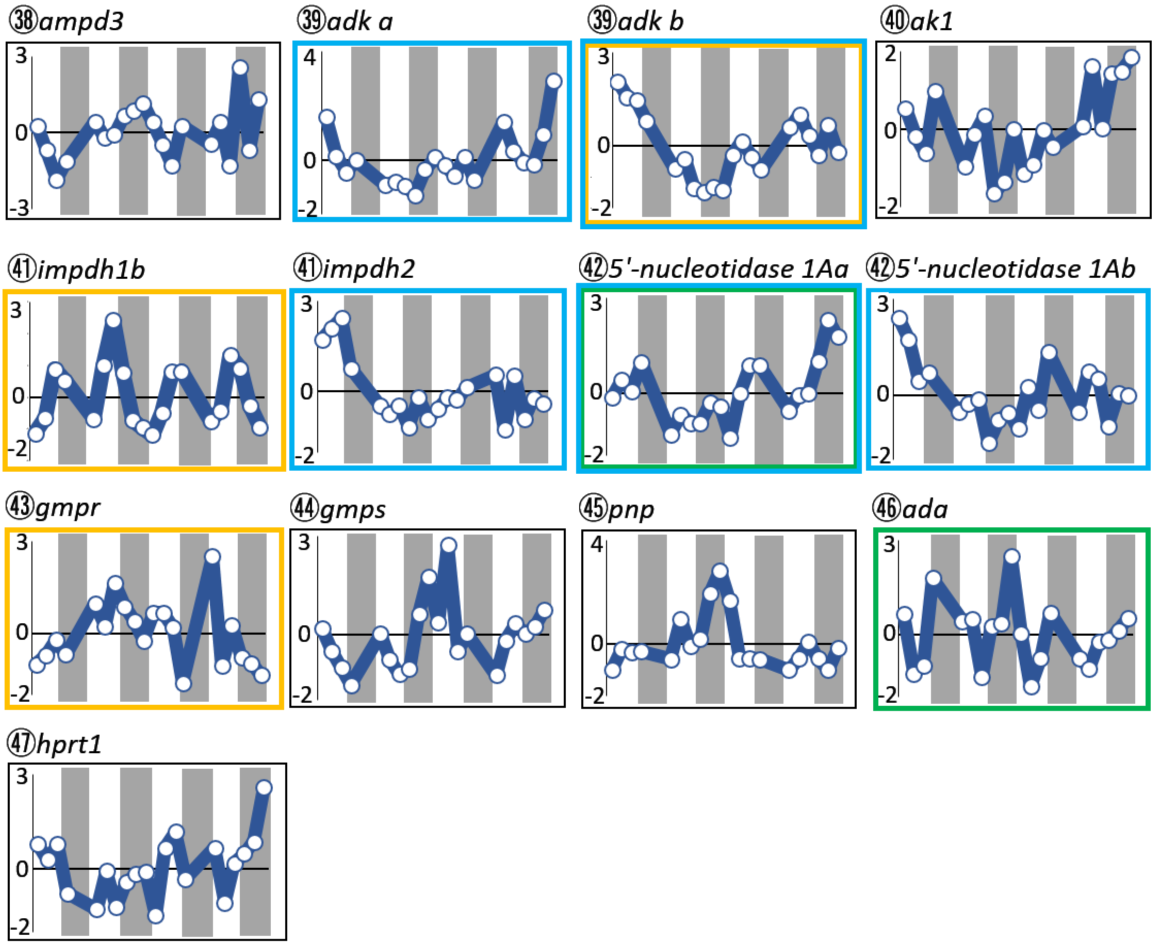

Fig. S13 The dynamics of gene expression on purine metabolism. Yellow and green colored gene exhibiting dinal and nocturnal fashion, respectively. Blue and pink showed the upregulation by fasting and feeding, respectively. Dark colored areas represent night. Encircled numbers were corresponded to figure 4. AMP deaminase (*ampd*), adenosine kinase (*adk*), adenylate kinase 1 (*ak1*), IMP dehydrogenase (*impdh*), GMP reductase (*gmpr*), GMP synthetase (*gmps*), purine nucleoside phosphorylase (*pnp*), adenosine deaminase (*ada*), and hypoxanthine phosphoribosyltransferase 1 (*hprt1*).

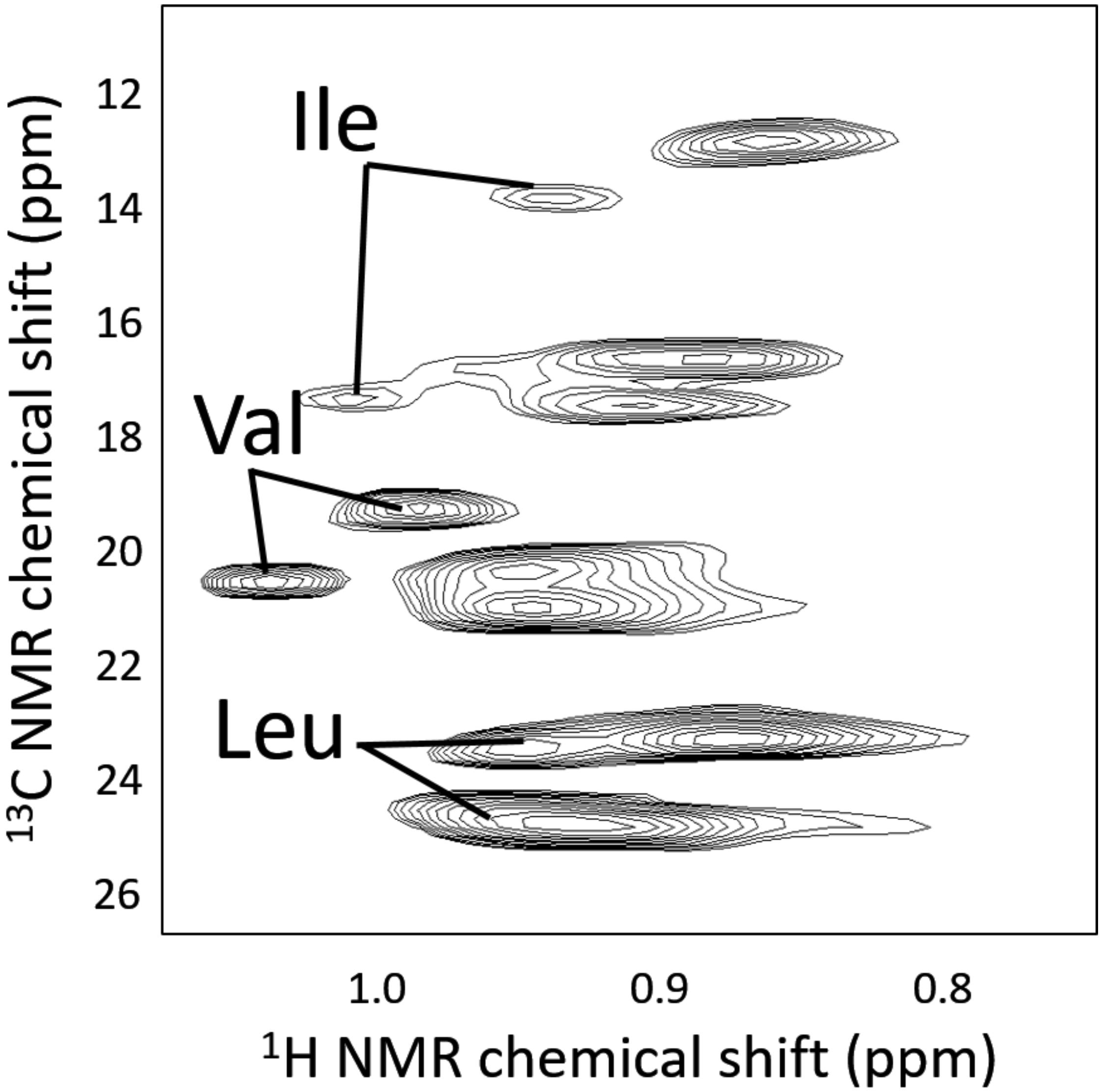

Fig.S14 Expanded methyl region of NMR spectra of feed.
^{1}H-^{13}C heteronuclear single quantum coherence (HSQC) NMR spectra of fish feed. Proton range (0.75-1.08 ppm), carbon range (10-26 ppm). Abbreviations are Isoleucine (Ile), valine (Val), and leucine (Leu).

Supplemental Table

Table S1 The top 100 highly expressed genes in muscle.

Table S2 Lists of clustered gene.

- Table S2A Lists of genes in cluster1.
- Table S2B Lists of genes in cluster2.
- Table S2C Lists of genes in cluster3

Table S3 Highly expressed genes in fasting(NF2) and feeding (F4).

- Table S3A Gene list of ZT2.
- Table S3B Gene list of ZT6
- Table S3C Gene list of ZT10.
- Table S3D Gene list of ZT14.
- Table S3E Gene list of ZT18.
- Table S3F Gene list of ZT22.

Table S4 Annotated metabolites detected in the HSQC-NMR spectra

Table S5 Lists of primers for qPCR validation
